# Semantic processing and individual suggestibility modulate motor preparation and perceived distance for looming sounds entering the peripersonal space

**DOI:** 10.1038/s41598-026-48067-4

**Published:** 2026-04-20

**Authors:** Roberto Barumerli, Michele Geronazzo, Paola Cesari

**Affiliations:** 1 Department of Neurosciences, Biomedicine and Movement, University of Verona, Verona, Italy; 2 Dyson School of Design Engineering, Imperial College London, London, UK; 3 Department of Industrial Systems Technology and Management, University of Padova, Vicenza, Italy

**Keywords:** Peripersonal space, Auditory looming stimuli, Spatial hearing, Anticipatory postural adjustments, Semantic modulation, Sensory suggestibility, Human behaviour, Information technology, Statistics, Auditory system, Emotion, Sensorimotor processing

## Abstract

Our brain maps the space immediately surrounding the body, the peripersonal space (PPS), to sharpen sensory-motor coordination whenever an object enters it. Within PPS, past research demonstrated how several factors influence motor readiness: from stimulus characteristics, such as body-object distance and stimulus semantics, to personality traits like anxiety. However, most paradigms infer PPS boundaries using tactile and visual stimuli, with auditory cues often playing an ancillary role, rather than directly measuring response modulation to stimuli entering the PPS. Here, we measured anticipatory postural adjustments, as direct physiological indices of motor planning, to examine whether semantic content and individual suggestibility modulate responses to looming sounds stopping within PPS. Thirty-three adults heard affective semantic sounds (positive - applause, negative - dentist drill) or neutral non-semantic (pink noise) sounds halting at five within-PPS distances while we recorded muscle activation timing, distance estimates, affective ratings, and sensory suggestibility. Motor responses were faster as sounds stopped nearer the body but systematically delayed and less precise for semantic sounds compared to non-semantic sounds, with higher suggestibility predicting longer and more variable latencies, particularly for non-semantic sounds. These findings demonstrate that semantic evaluation imposes measurable processing costs that systematically delay motor preparation and increase perceived distance. Individual suggestibility produces freeze-like response patterns that amplify motor uncertainty, specifically when semantic context is absent, revealing distinct cognitive and trait-based mechanisms that jointly regulate defensive behaviour within PPS.

**Supplementary Information:**

The online version contains supplementary material available at 10.1038/s41598-026-48067-4.

## Introduction

To successfully navigate the environment, the brain maintains a dynamic representation of the body’s surrounding space by integrating sensory perception with action planning^1^. This representation, known as peripersonal space (PPS), enables rapid responses to potential threats, such as sidestepping an unseen car, and facilitates goal-directed behaviours, like catching a buzzing mosquito in the dark. PPS serves as a sensorimotor interface for two distinct but related functions: (1) goal-directed (appetitive) actions, such as reaching and grasping objects we want to interact with, and (2) defensive responses to potential threats we need to avoid^2^. While appetitive actions require accurate spatial coding to guide the hand toward a target, defensive responses prioritise rapid detection and withdrawal, favouring speed over precision^3^. Despite these different demands, both functions share a common mechanism: the rapid integration of sensory information with motor preparation^1^. Because optimal response timing depends on context (e.g. reaching for a coffee cup requires different preparation than dodging a wasp), PPS boundaries dynamically adapt to both stimulus properties and individual interpretation of the stimulus. For instance, threatening stimuli enlarge PPS boundaries^4^, as do personality traits such as anxiety^5^, while greater interoceptive accuracy narrows them^6^. Prior methods characterise PPS through reachability judgments, skin conductance, hand-blink reflex, or event-related potentials^7^, but predominantly assess where boundaries lie rather than how perception-action coupling operates within them. The present study focuses specifically on the defensive function of PPS, investigating how the motor system prepares for potential impact from approaching (looming) stimuli entering the PPS, considering both stimulus properties and individual traits.

Looming objects provide an ecologically valid approach to studying perception-driven motor preparation within PPS, as they naturally signal potential threats, activating peripersonal neurons that respond to objects entering the PPS^2,8,9^ and engaging rapid sensorimotor transformations^10^. Across sensory modalities, stimuli approaching the body modulate motor system activity in a distance-dependent manner: visual objects approaching the hand enhance corticospinal excitability^11^, and sounds presented near the hand facilitate motor-evoked potentials^3,12^. Furthermore, sounds with rising intensity (i.e., the primary cue for approaching auditory sources^13^) presented diotically demonstrated to elicit a proximity change^14^ and activate motor and premotor cortices even without explicit motor tasks^15^. Because hearing cannot be blocked, covers space omnidirectionally, and responds faster than vision^16^, looming sounds represent an ideal stimulus for investigating defensive motor preparation^14^. Yet existing research has primarily employed looming sounds either to demonstrate faster and more accurate responses compared to receding sounds (i.e., looming bias)^17^ or to probe PPS boundary modulations, showing, for instance, that negatively valenced sounds expand the defensive space compared to neutral or positive sounds^4^. How looming sounds shape motor preparation as a function of proximity within PPS, rather than at its boundaries, remains largely unexplored, despite evidence that motor readiness scales continuously with proximity within PPS^18^. Addressing this question requires sounds that vary not only in distance but also in their processing demands. Semantic sounds (e.g., a dog barking vs. pink noise) require higher-order appraisal beyond localisation, engaging distinct neural mechanisms: spatial localisation (“where”) and content-based appraisal (“what”) operate through parallel streams^19^ on different timescales^20^. For approaching sounds, this temporal dissociation has behavioural consequences: during semantic evaluation, the stimulus continues moving, so spatial estimates reflect an earlier position: a ‘temporal tagging’ mechanism^21,22^ that affects both response timing and distance perception. Testing such temporal tagging effect requires measures that capture response timing with sufficient temporal resolution to detect processing-stage delays.

Anticipatory postural adjustments (APAs) offer a direct approach to investigate auditory-driven motor planning within PPS. APAs are early muscle activations, originating as feedforward commands from the premotor cortex^23,24^, that stabilise posture in preparation for movement. Although traditionally studied in voluntary goal-directed actions, recent evidence confirms that APAs are not limited to intentional movements; they are also an integral component of defensive, reactive responses to threats^18,25^. In this context, APAs serve as a ‘pre-emptive’ motor defence, reflecting how quickly the central nervous system initiates protective stabilisation before an actual impact or avoidance manoeuvre occurs. Therefore, their timing reflects how quickly the central nervous system processes a perceptual event and initiates motor preparation, regardless of whether the subsequent action is appetitive or protective^26^. Most importantly and unlike transcranial magnetic stimulation or tactile detection paradigms that infer PPS modulation from sensory facilitation^4^, APA measurement captures feedforward motor commands preceding natural movement^27^. Prior work shows that looming sounds stopping closer within PPS elicit earlier APA onset, whereas sounds stopping outside PPS produce no such modulation^18^. Additionally, our previous studies further demonstrated that semantic sounds (regardless of positive or negative valence) elicit delayed premotor reactions compared to non-semantic sounds^28^. This result suggests that the affective evaluation of meaningful auditory stimuli requires additional processing time, leading to a measurable latency in motor preparation^20^. However, existing work has focused on group-level effects, leaving open how individual differences modulate motor timing and variability within PPS.

Recent work investigating the link between perception and action shows its flexibility within PPS: sensory information is uncertain, therefore, people can actively interpret what they sense^29^. Individual differences in resolving this sensory uncertainty may determine the strength of sensorimotor coupling within PPS: as reported before, higher trait anxiety enlarges PPS boundaries^5^, whereas highly suggestible individuals show enhanced susceptibility to multisensory bodily illusions such as the rubber-hand illusion^30^, possibly because enhanced multisensory integration magnifies their responsiveness^31^. Although previous literature related PPS size to self-reported personality traits^32^, little is known about how traits affect the timing and variability of motor responses within PPS boundaries. Since PPS serves as a defensive interface requiring time-constrained translation of sensory signals into motor responses^2,33^, sensory suggestibility (i.e., heightened responsiveness to sensory and physiological cues^34^ should determine how readily individuals initiate motor preparation when proximity cues are ambiguous. This is particularly relevant for auditory distance perception, where distance must be decoded from inherently noisy acoustic features rather than from a topographic sensory map as in vision^13,35^. Looming sounds simulated through intensity modulation present ambiguous acoustic gradients that may engage individual suggestibility differences in interpreting proximity. Given the defensive function of PPS, this sensory uncertainty may manifest as a “freeze-like” response where highly suggestible individuals show increased motor hesitation or greater response variability before committing to defensive action^36^.

Building upon our previous finding that semantic processing induces a premotor latency^28^, the present study investigates how semantic content and individual sensory suggestibility jointly modulate the perception-action coupling for looming sounds. We combined motor planning measures (i.e., APAs) with an explicit distance estimation task to capture the interplay between physiological readiness and spatial perception. We compared responses to non-semantic sounds (pink noise) and to semantic sounds carrying distinct affective valences (positive: applause; negative: dentist drill). These stimuli were selected because they represent prototypical valence clusters (low and high valence with comparable arousal) within IADS normative ratings^37^ and lack discrete sub-events that could introduce temporal segmentation confounds. This design examines: (1) whether semantic versus non-semantic sounds engage different processing mechanisms, and (2) whether valence further differentiates responses within the semantic category. Participants heard looming sounds stopping at five simulated distances within PPS. Across two experimental sessions conducted one week apart, we measured four outcomes: APA timing and stopping distance estimates in the first, and affective ratings and suggestibility trait in the second, with motor, distance, and affective measures collected across all stimulus types and distance combinations. Affective properties were validated using the Self-Assessment Manikin (SAM)^38^, while the Physiological Reactivity (PHR) subscale of the Multidimensional Iowa Suggestibility Scale (MISS)^34^ quantified individual sensory suggestibility by measuring automatic bodily responses to sensory and emotional cues. We employed a Bayesian statistical framework to accommodate individual variability in reaction times and perceptual estimates^39^.

We formulated two main hypotheses. (H1) Semantic sounds will elicit delayed premotor responses and altered distance perception compared to non-semantic sounds. This prediction stems from the temporal hierarchy of auditory processing: spatial localisation (“where”) precedes semantic appraisal (“what”)^20^. While non-semantic sounds drive motor preparation via spatial cues alone, semantic sounds require additional ventral stream processing for affective evaluation. This necessary synchronisation introduces a processing latency (referred to as “semantic cost”) that is critical within a defensive PPS framework. Since defensive responses prioritise speed to ensure a safety margin, this delay may represent a functional vulnerability, as the stimulus continues its approach while the motor system awaits appraisal. Second, (H2) Individual differences in sensory suggestibility will account for variability in motor timing and its precision, particularly during the integration of spatial and semantic cues. As defensive PPS acts as a time-constrained interface, suggestibility likely determines how readily individuals initiate motor preparation when proximity signals are ambiguous.

## Results

With the general aim of investigating how auditory affective stimuli and individual suggestibility influence perceptual, spatial, and motor responses within the peripersonal space, we collected data from 33 participants (17 females; mean age = 23.3 ± 3.2 years) who completed three auditory tasks. MISS and SAM were administered in a separate session. Four participants did not attend this session, though their motor and distance estimation data remained complete. All participants self-reported normal hearing and no neurological or musculoskeletal impairments, gave written informed consent, and the study was approved by the Ethics Committee of the University of Verona (approved 30 March 2017).

**Fig. 1:**
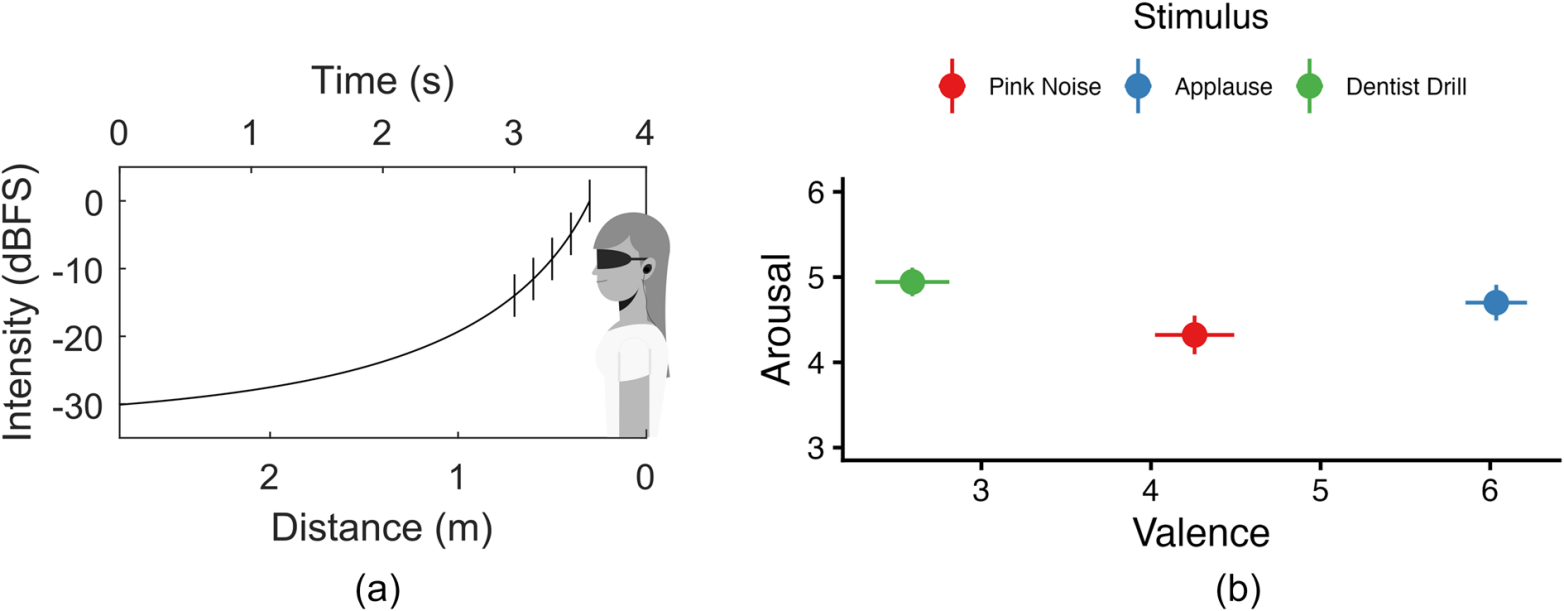
Characteristics of auditory stimuli and their affective evaluation. (**a**) Intensity profile simulating sound source approach via the inverse-square law over both time and simulated distance (depicted on top and bottom horizontal axes, respectively). Sounds were presented through headphones with diotic (non-spatialized) presentation which conveyed proximity change through rising intensity. Vertical bars indicate the five simulated stopping distances (0.3–0.7 m); zero represents the listener’s ear-canal position. (**b**) Scatterplot of mean arousal ratings as a function of mean valence ratings for three auditory stimuli (ratings were provided on a 9 points scale). Points and error bars represent means and standard errors corrected for within-subjects designs^42^.

The tasks assessed distinct perceptual and behavioural responses to three auditory stimuli: two affective sounds selected from the IADS2 database^37^(one with positive valence, ID 351 – Applause, and one with negative, ID 719 – Dentist Drill) and a neutral control stimulus (Pink Noise) without semantic content. Importantly, participants were never informed about stimulus identity nor asked to identify sounds. An amplitude envelope following the inverse-square law was applied to simulate approaching sound sources^13,40^, starting 2.8 m from the participant, moving at 0.7 m/s (walking speed^40^ and stopping at five different positions from participant ears (from 0.7 to 0.3 m in 0.1 m steps - see Fig. 1a). This velocity, consistent with our previous work^28,41^, avoids startle responses and allows measurement of anticipatory rather than reflexive postural adjustments. Similar effects at faster velocities^18^ suggest robustness across approach speeds. Importantly, stimuli were delivered through in-ear headphones and looming was simulated through diotic (non-spatialized) intensity increases, without binaural directional cues.

To integrate neurophysiological data, behavioural measures, and individual differences, we report results in three steps. First, we validated the affective evaluation of looming sounds to ensure clear categorical discrimination. Second, we employed a Bayesian hierarchical model to analyse distance estimates and muscle contraction timing and provide a rigorous account of how experimental factors jointly shape perception-action coupling within the PPS. This approach quantified population-level effects (i.e., with systematic biases as intercepts, sensitivity as slopes, and response variability as dispersion) while incorporating sensory suggestibility as a continuous predictor to model how this trait impacts response magnitudes and their residual variability across conditions.

### Affective evaluation of looming sounds

The affective evaluation was conducted using the SAM mannequin^38^, following the original methodology of the IADS2^37^, yielding strong concordance between our participants’ ratings and the original scores (see Fig. 1b). Given that categorical rating scales produce bounded, ordinal responses, we employed an Aligned Rank Transform (ART) ANOVA^43^ that revealed significant differences in valence across stimulus categories (F(2, 405) = 303.56, *p* < .001), but neither the distance factor (F(4, 405) = 1.99, *p* = .096) nor the interaction between stimulus category and distance showed significant effects (F(8, 405) = 0.61, *p* = .767). The partial eta-squared for the stimulus type was η_p_^2^ = 0.6. Post-hoc contrasts computed by employing ART-C tests with Tukey correction^44^ indicated that valence ratings differed significantly among all three sounds: negative (2.59 arbitrary unit (a.u.) ± 0.22) < neutral (4.26 a.u. ± 0.23) < positive (6.04 a.u. ± 0.18; all pairwise *p* < .001). Importantly, these differences in the valence score follow the intrinsic affective salience of the stimuli, as observed in the original work^37^, and how the noise stimulus resulted neutral in comparison to the other two stimuli^4^.

Similarly, statistical analysis of arousal values indicated significant main effects for both stimulus categories (F(2, 405) = 5.40, *p* = .005, η_p_^2^ = 0.03) and stopping distance (F(4, 405) = 5.09, *p* < .001, η_p_^2^ = 0.05), but not for their interaction (F(8, 405) = 0.63, *p* = .751). Post-hoc analysis on distance revealed that only the contrast between the nearest (0.3 m, mean ± standard error: 5.190 a.u. ± 0.135) and farthest (0.7 m, 4.083 a.u. ± 0.118) stopping positions reached significance (*p* < .001), with intermediate distances showing no reliable differences (*p* > .05). While perceived proximity modulates arousal through rising intensity cues^14^, this effect explained minimal variance in our within-PPS design. Post-hoc analysis further revealed that the positive stimulus (4.700 a.u. ± 0.209) did not differ from either neutral (4.321 a.u. ± 0.227) or negative stimuli (4.942 a.u. ± 0.169; all *p* > .05). The neutral stimulus was rated significantly lower in arousal than the negative stimulus (*p* = .003). Although statistically significant, the effect size for stimulus type was small and the small magnitude difference (0.621a.u. ± 0.362 points on a 9-point scale) suggests comparable arousal across stimuli. Further, all three sounds showed tightly clustered arousal ratings (4.65 a.u. ± 0.96), which was a much narrower spread than the full IADS arousal spectrum (6.23 a.u. ± 2.16). Together, these minimal effect sizes and small absolute differences indicate that arousal remained sufficiently stable across experimental conditions to isolate the effects of semantic content and distance on motor responses.

### Suggestibility questionnaire

Twenty-nine participants completed the Physiological Reactivity (PHR) subscale from the MISS questionnaire, a 13-item measure^34^. We used the PHR measure of self-assessed sensory suggestibility to evaluate the tendency to accept and act on perceived physiological states with reduced critical evaluation. Higher PHR scores indicate greater suggestibility, defined as a stronger propensity to experience physiological responses to sensory and emotional cues (e.g., heart pounding when thinking about something scary), while lower scores reflect more independent judgment of such cues. The subscale demonstrated good internal consistency (i.e., all items contributed coherently to the same construct), yielding Cronbach’s α = 0.810 with a 95% confidence interval of [0.581, 0.897]. Further, the distribution of raw scores was approximately normal (Shapiro-Wilk test, *p* = .654); therefore, we standardised the scores by subtracting the sample mean and dividing by the sample standard deviation.

### Distance estimation of looming sounds

**Fig. 2:**
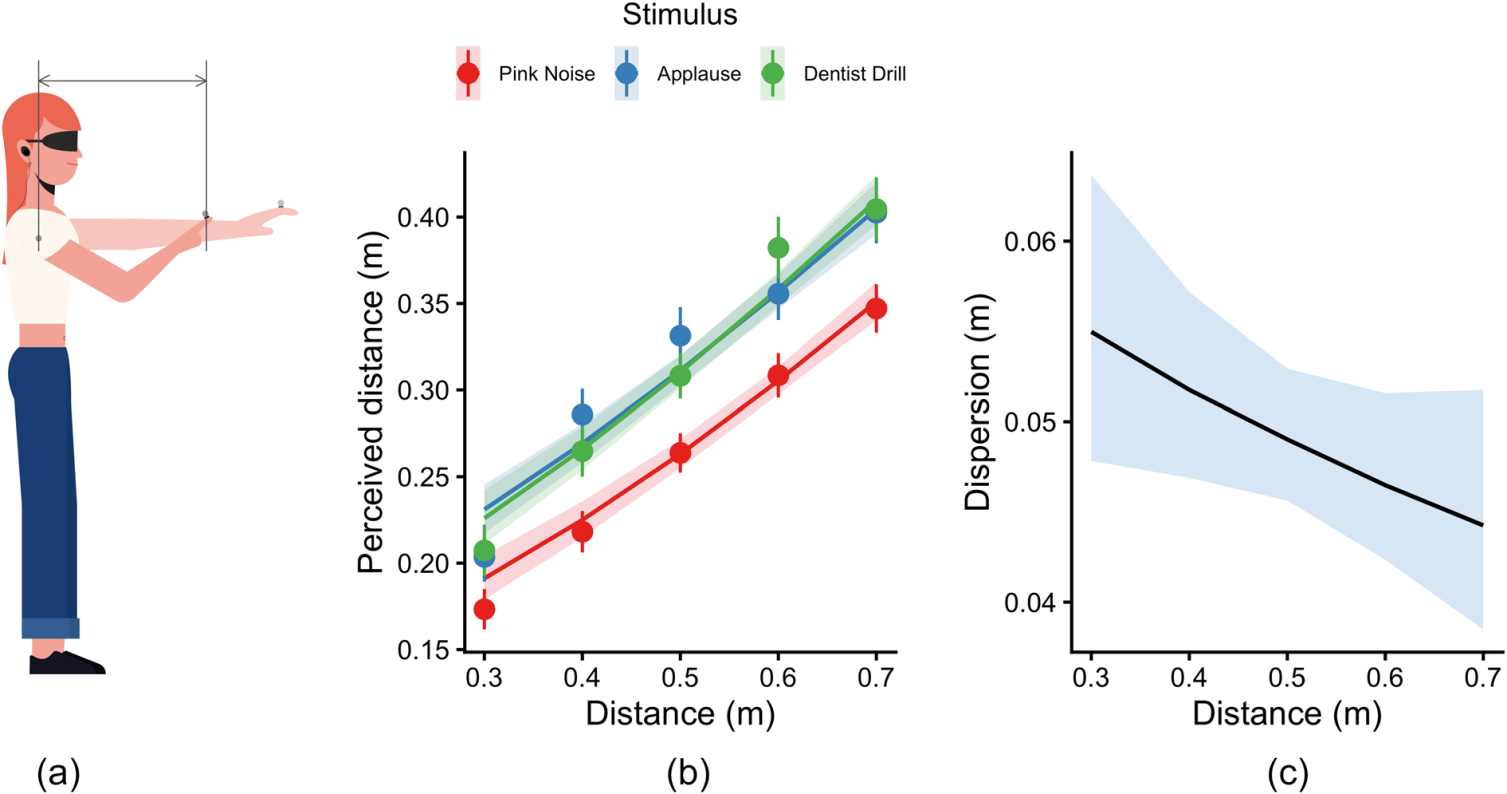
Distance estimation task and perceived distance of looming sounds. (**a**) Visualisation of a participant using their arm to provide the perceived final distance of the presented stimulus. (**b**) Estimated response of distance as a function of simulated ending distance for three auditory stimuli (Pink Noise, Applause, and Dentist Drill). Shaded areas represent 95% credible intervals of Bayesian beta regression while points and error bars report mean and standard errors computed over participants corrected for within-subjects designs^42^. (**c**) Response dispersion (i.e., standard deviation) as a function of distance, displayed with credible intervals at 95% level.

Blindfolded participants estimated the stopping distance by pointing along their extended dominant arm to indicate where the sound stopped (Fig. 2a), a body-centred measure appropriate for near-field auditory localisation within PPS (refer to Methods for details). Our analytical approach first utilized a preliminary factorial analysis to identify global effects, followed by a Bayesian hierarchical model to characterize specific parameters.

Given the non-normal distribution of bounded responses, we conducted a repeated-measures ART-ANOVA on within-subject normalised (z-scored) distances. The analysis on the full sample (*N* = 33) revealed significant main effects of distance (F(4, 480) = 381.57, *p* < .001, η_p_^2^​ = 0.76), stimulus type (F(2, 480) = 9.75, *p* < .001, η_p_^2^​ = 0.27), and their interaction (F(8, 480) = 2.88, *p* = .004, η_p_^2^​ = 0.05). To characterize these patterns while integrating individual traits, we report the parameters from the fitted Bayesian beta regression model with weakly informative priors (*N* = 29) (refer to Methods 4.3 for details), which explained a substantial proportion of the variance in distance judgments (Bayesian R^2^ = 0.77, 95% credible interval (95%-CI) [0.74, 0.79]). For completeness, we reported post-hoc analysis in the supplementary materials.

The Bayesian model confirmed the ART-ANOVA findings (see Table 1; Fig. 2b): semantic sounds were perceived as stopping further from the body than neutral noise (positive: +0.048 m, probability of direction (pd) = 1.00; negative: +0.047 m, pd = 1.00). This systematic overestimation indicates that identifiable auditory objects are perceived as more distal than abstract noise, regardless of their specific valence, as evidenced by the lack of credible difference between positive and negative marginal means (pd = 0.51). The model also confirmed a high degree of spatial sensitivity, with perceived distance scaling linearly with simulated proximity (pd = 1.00). While discrimination ability (i.e., the slope) was generally consistent across stimuli, negative sounds showed a weak increase in distance sensitivity compared to neutral noise (pd = 0.95), whereas positive sounds did not differ from baseline (pd = 0.83). This divergence accounts for the interaction observed in the frequentist analysis.

In contrast to stimulus manipulations, sensory suggestibility (PHR) did not predict overall distance estimates (pd = 0.59). However, PHR increased distance sensitivity for neutral sounds (0.066 m/m per standardized PHR unit, 95%-CI [0.002, 0.122], pd = 0.98); this effect was abolished for semantic sounds, with negative sounds showing weak attenuation (− 0.074 (m/m)/a.u, pd = 0.95) and positive sounds trending similarly (− 0.047 m/m, pd = 0.87). This suggests that semantic processing eliminated trait-dependent modulation of spatial discrimination.

**Table 1:** Bayesian beta regression model parameters for perceived distance: effects of stimulus type, distance, and sensory suggestibility (PHR) on mean estimates and response dispersion.

Submodel		Mean (µ)			Dispersion (σ)		
Section	Parameter	Estimate	95% CI	p(d)	Estimate	95% CI	p(d)
Marginal mean (m)	Pink noise	0.267	[0.259, 0.276]	**1.00**	0.043	[0.037, 0.051]	**1.00**
	Positive	+ 0.048	[0.036, 0.059]	**1.00**	+ 0.010	[-0.002, 0.021]	**0.95**
	Negative	+ 0.047	[0.035, 0.059]	**1.00**	+ 0.009	[-0.003, 0.021]	0.94
Slopes: distance (m/m)	Pink noise	0.399	[0.352, 0.445]	**1.00**	-0.036	[-0.082, 0.009]	**0.95**
	Positive	+ 0.035	[-0.037, 0.105]	0.83	+ 0.024	[-0.044, 0.092]	0.76
	Negative	+ 0.060	[-0.01, 0.131]	**0.95**	+ 0.005	[-0.058, 0.068]	0.56
Slopes: PHR (m/a.u.)	Pink noise	0.003	[-0.023, 0.029]	0.59	-0.004	[-0.014, 0.006]	0.78
	Positive	-0.003	[-0.017, 0.012]	0.64	+ 0.011	[-0.004, 0.025]	0.92
	Negative	+ 0.006	[-0.007, 0.021]	0.81	+ 0.005	[-0.01, 0.02]	0.75
Cross-slopes: distance × PHR ((m/m)/a.u.)	Pink noise	0.066	[0.002, 0.122]	**0.98**	-0.009	[-0.062, 0.043]	0.63
	Positive	-0.047	[-0.124, 0.037]	0.87	+ 0.062	[-0.013, 0.139]	**0.95**
	Negative	-0.074	[-0.159, 0.013]	**0.95**	+ 0.015	[-0.062, 0.092]	0.66

Marginal effects computed from posterior predictions. Effects computed for positive and negative sounds are in relation Pink Noise (i.e., baseline). CI = 95% credible interval. p(direction) = probability of direction.

Dispersion computed as σ = √[μ(1-μ)/(1+φ)] with μ being mean perceived distance and φ being the beta distribution’s precision parameter. Positive values indicate increased variability.

### Response uncertainty

The Bayesian model allowed us to analyse response dispersion (standard deviation, Fig. 2c; Table 1) across experimental factors. Baseline dispersion was 0.043 m (95%-CI [0.037, 0.051]), and semantic sounds showed anecdotally higher dispersion than neutral noise (positive: pd = 0.95; negative: pd = 0.94). Further, the model demonstrated that response dispersion decreased as sounds stopped further from the body for neutral sounds (pd = 0.95), with no credible difference in this distance-dispersion relationship for semantic stimuli (positive: pd = 0.76; negative: pd = 0.56). Individual suggestibility (z-scored PHR) did not reliably modulate overall dispersion (pd = 0.78). However, for positive sounds, higher suggestibility was associated with both an increasing trend for dispersion (pd = 0.92) and a steeper distance-dispersion slope (pd = 0.95), suggesting that suggestible individuals showed greater and more distance-dependent variability for positively-valenced stimuli. Overall, response precision was primarily governed by acoustic distance, with secondary contributions from semantic content and, for positive sounds specifically, individual suggestibility.

### Premotor reaction time (pm-RT)

**Fig. 3:**
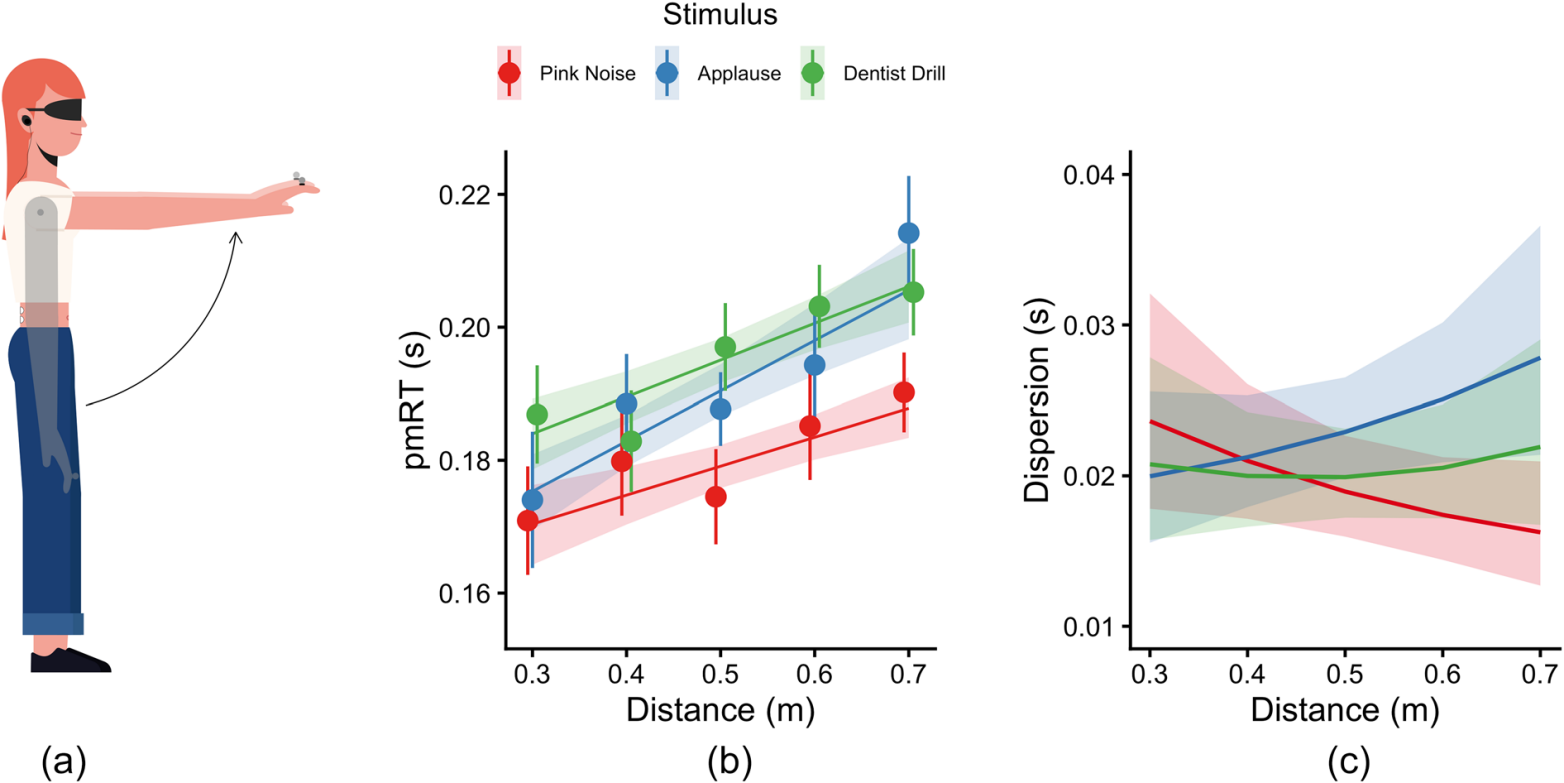
Premotor reaction time (pmRT) and its timing variability for looming sounds. (**a**) Visualisation of the participant raising their arm after the stimulus presentation. (**b**) Premotor reaction times as a function of simulated ending distance for three auditory stimuli (Pink Noise, Applause, and Dentist Drill). Shaded areas represent 95% credible intervals of Bayesian regression and points and error bars representing mean and standard errors over participants corrected for within-subjects designs^42^. (**c**) Timing dispersion (i.e., standard deviation) as a function of distance, displayed with credible intervals at 95% level.

In this task, we asked participants to raise their arms as quickly as possible after the perceived end of the looming sound to measure postural adjustments as the contraction initiation of postural muscles^18^ (as shown in Fig. 3a). We derived the premotor reaction time (pm-RT) as the activation time of the erector spinal muscles. Following the same analytical approach as distance estimation, we first conducted a factorial analysis followed by Bayesian hierarchical modelling.

The analysis of variance (ANOVA) on data z-scored per participant (*N* = 33) demonstrated significance for main effects of stimulus type (F(2, 480) = 90.754, *p* < .001, η_p_^2^​ = 0.29) and distance (F(4, 480) = 381.571, *p* < .001, η_p_^2^​ = 0.37), and their interaction (F(8, 480) = 2.881, *p* < .004, η_p_^2^​ = 0.10). We further investigated the trends using a Bayesian hierarchical linear model (see “Apparatus”), which extended the ANOVA findings (see Fig. 3b), and explained a substantial portion of the motor response variance (Bayesian R^2^ = 0.72, 95%-CI [0.67, 0.76]).

The model indicated a robust linear relationship between sound stopping position and motor timing (see Table 2; Fig. 3b), where pm-RT increased as sounds stopped further from the body (pd = 1.00). Crucially, timing of muscle contraction was significantly modulated by stimulus type: both semantic sounds induced comparable motor delays compared to pink noise (positive: +0.011 s, pd = 1.00; negative: +0.016 s, pd = 1.00) with negative sounds producing greater delays than positive (+ 0.005 s, 95%-CI [0.000, 0.009], pd = 0.97). Additionally, positive sounds elicited a steeper distance-response slope (pd = 0.97), indicating increased sensitivity to the proximity of positively-valenced content. In contrast, negative sounds showed no credible change in distance sensitivity (pd = 0.78).

**Fig. 4:**
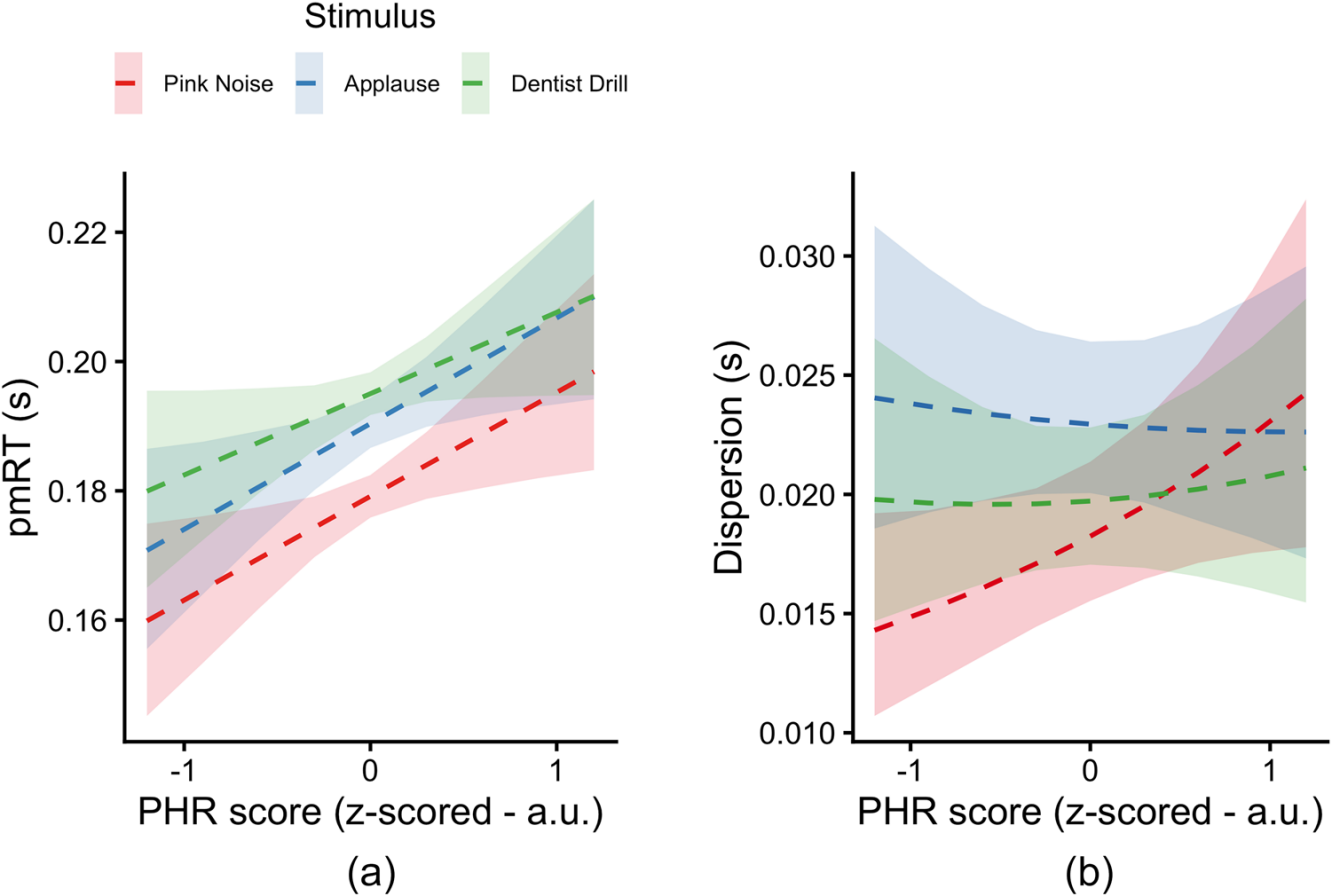
Influence of suggestibility on premotor reaction time and its dispersion. (**a**) The plot shows the linear relationship between normalised PHR score and pm-RT response for three stimulus categories. (**b**) The plot reports the effect of the normalised PHR score on response dispersion, which is shown separately for three categories. Lines represent linear regressions, and shaded areas indicate the 95% credible intervals of slope estimates. PHR score is reported on a normalised scale (arbitrary unit – a.u.).

Beyond experimental factors, individual sensory suggestibility (PHR) emerged as a robust predictor of motor timing (Fig. 4a). The Bayesian model revealed that higher suggestibility scores were associated with a global slowing of motor responses (pd = 0.99), consistent with a “freeze” response during the evaluation of looming stimuli. This trait-dependent slowing was largely consistent across stimulus types, with no credible difference for positive sounds (pd = 0.54) and only weak attenuation for negative sounds (pd = 0.95). The Distance × PHR interaction did not differ credibly across stimulus types (all pd ≤ 0.90), indicating that while suggestibility affects overall motor timing, it does not modulate distance sensitivity. Together, these results demonstrate that suggestibility induces baseline delays in motor preparation, while the underlying spatial processing within peripersonal space remains preserved.

**Table 2:** Bayesian linear regression model parameters for premotor reaction time: effects of stimulus type, distance, and sensory suggestibility (PHR) on mean timing and response dispersion.

Submodel		Mean (µ)			Dispersion (σ)		
Section	Parameter	Estimate	95% CI	p(d)	Estimate	95% CI	p(d)
Marginal mean (s)	Pink noise	0.179	[0.176, 0.182]	**1.00**	0.019	[0.016, 0.024]	**1.00**
	Positive	+ 0.011	[0.006, 0.016]	**1.00**	+ 0.004	[-0.001, 0.009]	0.92
	Negative	+ 0.016	[0.011, 0.021]	**1.00**	+ 0.001	[-0.004, 0.006]	0.67
Slopes: distance (s/m)	Pink noise	0.044	[0.023, 0.065]	**1.00**	-0.017	[-0.041, 0.002]	**0.96**
	Positive	+ 0.032	[0, 0.066]	**0.97**	+ 0.037	[0.007, 0.072]	**0.99**
	Negative	+ 0.011	[-0.017, 0.042]	0.78	+ 0.021	[-0.009, 0.052]	0.92
Slopes: PHR (s/a.u.)	Pink noise	0.016	[0.004, 0.028]	**0.99**	0.004	[0, 0.009]	**0.98**
	Positive	+ 0.000	[-0.004, 0.005]	0.54	-0.005	[-0.01, 0]	**0.97**
	Negative	-0.004	[-0.007, 0.001]	**0.95**	-0.004	[-0.01, 0.002]	0.90
Cross-slopes: distance × PHR ((s/m)/a.u)	Pink noise	0.010	[-0.007, 0.027]	0.88	-0.017	[-0.045, 0.003]	0.94
	Positive	-0.019	[-0.049, 0.011]	0.90	+ 0.002	[-0.034, 0.039]	0.54
	Negative	-0.004	[-0.034, 0.022]	0.62	-0.008	[-0.044, 0.03]	0.66

Marginal effects computed from posterior predictions. Effects computed for positive and negative sounds are in relation Pink Noise (i.e., baseline). CI = 95% credible interval. p(direction) = probability of direction.

Dispersion computed as standard deviation of residuals. Positive values indicate increased variability.

### Timing variability

Analysis of motor response variability (standard deviation) revealed that timing precision was shaped by stimulus type, its stopping distance and individual suggestibility (see Table 2).

The Bayesian model indicated that baseline variability for pink noise was 0.019 s (95%-CI [0.016, 0.024]), with positive sounds showing marginally higher dispersion (pd = 0.92) while negative sounds did not differ from baseline (pd = 0.67) (Fig. 3c). Instead, distance exerted opposite effects on variability depending on stimulus properties. For pink noise, dispersion showed a tendency to decrease with stopping distance (pd = 0.96), suggesting slightly more consistent timing for abstract sounds as they stop further from the body. Positive sounds reversed this pattern, showing increased dispersion with distance (+ 0.037 s/m vs. baseline, pd = 0.99), while negative sounds anecdotally neutralised the baseline effect (+ 0.021 s/m, pd = 0.92), resulting in no distance-dependent change in variability. This suggests that semantic processing disrupts the distance-precision relationship observed for abstract sounds.

Individual suggestibility (PHR) further modulated timing precision in a stimulus-dependent manner (Fig. 4b). For non-semantic pink noise, higher suggestibility increased response variability (pd = 0.98), with weak evidence that this effect diminished at farther distances (Distance × PHR: pd = 0.94). This PHR effect was attenuated for semantic sounds (positive: pd = 0.97; negative: pd = 0.90), effectively eliminating the PHR-dispersion relationship when sounds carried semantic content, with no Distance × PHR interaction (positive: pd = 0.54; negative: pd = 0.66). In sum, while suggestibility degraded timing precision for abstract sounds, semantic content appeared to stabilize motor variability across individuals.

## Discussion

We investigated how semantic content and individual suggestibility jointly shape proximity perception of looming sounds entering the PPS, combining physiological measurements, behavioural responses, and self-assessed questionnaires within a simulated auditory environment. Our findings reveal both expected patterns and novel dissociations. As expected, closer stopping distances produced shorter perceived distances and faster motor responses. Instead, semantic content and individual suggestibility exerted distinct influences on sensorimotor integration within PPS. We confirmed the first hypothesis (H1): semantic sounds (Applause and Dentist Drill) were systematically perceived as more distant and elicited slower motor responses compared to non-semantic pink noise, indicating that semantic extraction imposes processing costs on both spatial perception and motor preparation. The second hypothesis (H2) was partially supported with an unexpected dissociation: individual suggestibility significantly modulated distance estimations and motor timing, but only for non-semantic stimuli, where higher suggestibility increased distance sensitivity for pink noise. Similarly, suggestibility degraded timing precision for pink noise but not for semantic stimuli, indicating that semantic processing masked trait-dependent modulation of auditory-motor coupling. Following, we discuss the results in more detail.

In this study, we relied on auditory distance perception to demonstrate that individualised looming sounds effectively modulate distance estimation and APA timing within the PPS. Our stimuli simulated approach through diotic intensity modulation following the inverse-square law, prioritising intensity as the primary auditory distance cue^45^. While the scientific literature indicates that additional cues, such as reverberation or spectral information, enhance ecological validity^13^, we deliberately employed this simplified rendering to isolate the contribution of intensity-based looming to motor preparation. The results confirmed that this approach was sufficient for both behavioural and neurophysiological measures. In the distance estimation task, participants correctly discriminated stopping distances, demonstrating their ability to decode proximity from rising intensity. Similarly, pm-RT measurements successfully replicated established patterns where sounds stopping nearer the body elicit faster reactions^18,28,41^. While stimulus duration covaried with intensity in our design, prior work demonstrated that flat-envelope sounds with modulated duration do not elicit pm-RT modulation, indicating that duration alone does not create the percept of approach^18,46^. These validations confirm that our simplified rendering provided a controlled foundation for examining how semantic content modulates auditory-motor coupling beyond basic proximity processing.

Extending previous findings, our study examined how semantic content influences responses within PPS. If valence alone governed these responses, pink noise, with its intermediate affective rating, should produce intermediate motor timing and distance estimates. Instead, we observed a clear dissociation: pink noise elicited the fastest reactions and closest distance estimates, while both semantic sounds (regardless of positive or negative valence) produced comparable delays and distance overestimation. In addition, the observed slope differences across stimulus types were inconsistent between measures, suggesting these reflect stimulus-specific properties rather than a systematic processing time × distance confound. These patterns indicate that semantic identification, not affective content per se, imposes processing cost. The temporal hierarchy of auditory processing supports this interpretation: spatial localisation (“where”) proceeds more rapidly^20^ than semantic identification (“what”)^47^, with affective evaluation also engaging additional amygdala-auditory cortex circuitry^48^. Acoustic artefacts cannot explain our results: intensity-based looming alone elicits approaching detection^49^, and the pattern replicated with free-field loudspeaker presentation^28^, arguing against reverberation confounds. The consistent pattern across both measures suggests a common processing cost, though our design cannot determine whether these effects share a causal relationship or arise independently from the same bottleneck. One candidate mechanism is temporal tagging^21,22^: since semantic identification (“what”) is slower compared to spatial localisation (“where”) in auditory dual-stream processing^20^, the central nervous system must integrate these asynchronous streams at decision time. Because the sound continues advancing during semantic processing, the distance estimate might reflect an earlier evaluation from when the source was still moving closer. Such a mechanism parsimoniously predicts that semantic sounds are perceived as more distant, consistent with our results.

A potential confound for the temporal-tagging interpretation deserves explicit consideration. Arousal and semantic evaluation covary in real-world acoustic stimuli and cannot be fully disentangled, a limitation explicitly acknowledged in the development of affective auditory stimulus databases^50^. However, the data argue against an arousal-driven account on three grounds. First, arousal ratings were comparable across stimuli, with stimulus type explaining negligible arousal variance compared to valence. Second, the negative stimulus, which was rated marginally highest in arousal, produced slower motor responses than pink noise (i.e., the opposite of what heightened arousal would predict). Third, proximity-dependent arousal modulation was equivalent across sound types, ruling out a distance-arousal confound. Together, these converging lines of evidence favour semantic processing cost as the operative mechanism.

Beyond the established role of semantic content in shaping PPS responses, our data revealed that individual differences in sensorimotor integration are systematically modulated by trait suggestibility. Participants with higher suggestibility showed slower reactions across all sound types, consistent with an active ‘freeze’ response that facilitates threat assessment and response selection^36^. This freeze pattern aligns with neurophysiological evidence that approaching sounds engage threat-related brain networks, including the amygdala^10^ and frontal control regions^51^, which may facilitate threat assessment before initiating defensive action. Within the “last-second” defensive framework^52^, highly suggestible individuals may experience enhanced or prolonged engagement of these evaluative processes before committing to action. Critically, this suggestibility effect interacted with stimulus semantics: while semantic sounds (positive and negative) uniformly slowed responses regardless of individual differences in variability, pink noise revealed a distinct pattern where high suggestibility predicted both slower and more variable motor timing. This dissociation suggests that when semantic content engages the ventral auditory stream for identification^53^, it constrains motor preparation timing despite individual differences in suggestibility. Conversely, for non-semantic sounds relying mostly on dorsal stream spatial processing, suggestibility amplifies uncertainty in the perception-action coupling. Such results indicate that increased suggestibility affects top-down modulation differently depending on whether semantic context is available, potentially through differential pre-frontal cortex modulation^26,54^ that produces consistent motor timing for semantic sounds but leaves non-semantic sounds subject to individual variability.

While the defensive-oriented role of PPS has been thoroughly explored, our results extend previous findings by demonstrating that response dispersion measures are also significantly modulated in the proposed within-PPS experimental design. Specifically, we observed that the presence of semantic content interacted with individual suggestibility, systematically influencing both response timing and precision. Recent studies have indicated that predictability and emotional context can alter the sharpness or variability of PPS boundaries^55,56^. In line with these observations, our data revealed that stimuli free of semantic meaning elicited increased variability in participants with higher suggestibility, suggesting broader or less precise PPS representations when meaningful contextual cues are absent. Conversely, when semantic context was present, response variability remained consistently higher irrespective of suggestibility, potentially due to additional cognitive processing demands related to decoding stimulus meaning and emotional significance^57^. This aligns with neural findings indicating high dorsal auditory stream engagement under conditions requiring explicit spatial evaluation without semantics^20^. Importantly, this modulation of residual dispersion emerged primarily in the pm-RT measure and not in the cognitively mediated estimated-distance task, likely because the slower, deliberate pointing response allowed participants more cognitive control, reducing perceived threat and urgency^58^.

Despite the extensive study of PPS using visual and tactile inputs, the potential of auditory stimuli, especially in modulating motor readiness and spatial perception, remains an open and promising avenue for investigation. Our study highlights that auditory information alone can effectively shape perception-action coupling within PPS representations. Future research should further refine auditory simulations in two key directions. First, systematic variation of approach velocity would clarify how speed modulates perception-action coupling within PPS. Second, introducing ecologically valid neutral sounds incorporating realistic spectral and binaural cues beyond intensity changes would better approximate real-world acoustic dynamics^13,59^ and provide insights into the perceptual dimension of auditory distance processing. Moreover, given the known interactions between interoceptive accuracy and PPS boundaries^6^, it would be valuable to integrate physiological measures (e.g., heartbeat tracking) into our paradigm to examine how individual differences in interoceptive sensitivity modulate auditory PPS processing and pm-RT. Additionally, our personalised auditory rendering approach lends itself naturally to implementations within immersive virtual reality^41^. Within these immersive scenarios, future studies could systematically investigate not only the multisensory integration of visual and auditory stimuli but also how imaginative suggestibility and sense of presence influence PPS boundary plasticity^60^. Exploring emotional processing further, dynamic measures of postural control^61^ alongside pm-RT and PPS measurements could clarify how affective auditory stimuli shape behaviours (e.g., freezing or avoidance) within PPS^62^. Lastly, future investigations through neuroimaging methods, such as connectivity measures^51^, could examine how auditory “what” and “where” auditory pathways^20^ interact to modulate sensorimotor integration in response to looming auditory stimuli^47^. Importantly, our modelling approach based on Bayesian statistics can readily integrate novel measures for exploring how individual differences (e.g., personality, anxiety, interoceptive sensitivity) and stimulus properties jointly determine the variability in these neural and behavioural responses^63^, offering deeper insights into the underlying mechanisms shaping PPS representations.

## Conclusions

Our findings demonstrate that auditory looming stimuli within the PPS effectively modulate postural adjustments and distance estimation, influenced by both auditory distance, semantic content and individual suggestibility. We confirmed that stopping distances impacted our measures: as sounds stopped farther within the PPS, participants responded more quickly and estimated the distance to be closer to the body. Instead, semantic information impacted experimental measures, slowing motor responses and increasing the perceived auditory stopping distance, likely reflecting additional neural processing demands to address the stimuli’s semantic content. Notably, individual suggestibility shaped both reaction timing and its dispersion, highlighting the importance of considering internal personality traits when studying sensorimotor integration within the PPS. These findings reinforce the dynamic and personalised nature of PPS representations, extending our understanding of how the human brain integrates sensory, cognitive, and personality-driven processes to organise defensive and motor behaviours in response to approaching stimuli. In doing so, our study extensively explores the suggestibility role in sensorimotor integration with the aim of providing a blueprint for future research linking other personality factors, such as the Big Five personality traits^64^, to perception-action coupling within the PPS.

## Methods

### Participants

Thirty-three right-handed adults (17 women; mean age: 23.4 ± 3.15 years) with self-reported normal hearing and no known neurological or musculoskeletal impairments volunteered for the study. Before participation, written informed consent was obtained from each individual. The study protocol was approved by the Ethics Committee of the Department of Neurosciences, Biomedicine, and Movement Sciences at the University of Verona (approved 30 March 2017), and all procedures were carried out in accordance with the Declaration of Helsinki.

### Stimulus generation

Three auditory stimuli were selected from the International Affective Digitised Sounds (IADS) database, which provides standardised ratings of valence and arousal^37^. A single stimulus belongs to one affective category: Applause (positive valence - IADS ID 351), Dentist Drill (negative valence - IADS ID 719), and Pink Noise (neutral control). The neutral stimulus, Pink Noise, was synthetically generated using MATLAB’s audio toolbox. Each stimulus was band-pass filtered (0.25–9.5 kHz) and RMS amplitude-normalised using the Pink Noise level as a reference to ensure consistent loudness and spectral content across stimuli.

To simulate dynamic proximity, all sounds were transformed into looming stimuli through amplitude modulation by applying the inverse-square law of sound intensity decay, producing an exponentially rising intensity that mimicked a sound source approaching the body^40^. Specifically, the stimulus intensity $\:I$ followed the physical relation $\:I\:\propto\:\:\frac{1}{{d}^{2}}$, with $\:d$ being the simulated distance between the source and the listener.

Each looming sound began at a simulated distance of 2.8 m, maintained a constant velocity of 0.7 m/s, and stopped at one of five predefined simulated endpoints within the auditory PPS (0.3, 0.4, 0.5, 0.6, and 0.7 m)^18,41^. The corresponding sound level increased from 65 dBA to 95 dBA across the trajectory^41,46^. A 15-ms raised cosine onset ramp and 20-ms offset ramp were applied to each stimulus to suppress acoustic startle responses and avoid off-response artefacts.

Since looming was simulated solely through intensity, our stimulus processing effectively suppressed reverberant cues through multiple mechanisms: diotic presentation eliminated the interaural decorrelation necessary for spatial perception of reverberation; truncating all sounds to identical durations removed reverberant tails and late reflections carrying room acoustic information; the imposed looming envelope reduced temporal envelope features from original recording environments, rendering any residual reverberant cues perceptually unavailable.

### Apparatus

The experimental procedures were conducted in the Biomechanics Laboratory of the Department of Neurological, Biomedical and Movement Sciences, University of Verona. Motion data were collected using a VICON MX Ultranet motion capture system (Oxford Metrics, UK), comprising eight Vicon MX13 cameras operating at a 250 Hz sampling rate. Reflective markers were placed on the head (midsagittal plane), shoulders, and index fingers to record postural and gestural kinematics. Electromyographic (EMG) data were acquired from the erector spinae (ES) muscles using the ZeroWire EMG system (Aurion, Italy), with a sampling rate of 2000 Hz. The EMG multichannel analogue output was synchronised with the motion capture system via the Vicon MX control interface. Custom MATLAB scripts enabled the automation of the experimental procedure and data collection.

Auditory stimuli were diotic (non-spatialized) and delivered through Hefio One in-ear headphones, a research prototype featuring individualized ear canal calibration to equalize spectral transmission and ensure consistent intensity profiles across participants (refer to Geronazzo et al. 2023 for technical specifications^41^). An external audio interface (Saffire LE, Focusrite, UK) was used to amplify the audio signal. Sound intensity was calibrated using a CESVA SC-2c sound level meter, and playback was synchronised with kinematic data via the Vicon system. The Vicon MX control interface guaranteed synchronised recording of the audio signal with the motion capture and EMG data streams.

### Procedure

Data collection occurred across two experimental sessions. In the first session, participants completed the Reactive Task (pm-RT) followed by the Auditory Distance Estimation task in fixed order. The second session, conducted approximately 2 weeks later, comprised the Affective Evaluation (SAM) and the MISS questionnaire. We collected SAM ratings in a separate session to prevent repeated stimulus exposure from biasing participants’ affective evaluations, while trait suggestibility (MISS) was assumed stable over this time interval. Four participants did not attend the second session, resulting in missing SAM and MISS data for these individuals.

### Task 1: reactive task

Participants were positioned at the centre of the recording space and blindfolded throughout the entire task execution to reduce visual interference in the responses. They sat upright with their hands resting comfortably along their sides. With this task, we measured APAs to quantify the timing of feedforward control in action anticipation, i.e., changes in muscle activation that occur before the initiation of a voluntary movement^65^. To elicit APAs, participants were instructed to quickly raise their arms forward in response to the cessation of each sound, triggering postural perturbations caused by dynamic intersegmental forces that shift the body’s center of mass forward, thus requiring preparatory muscle adjustments to maintain vertical posture. Auditory stimuli, designed to simulate looming sound sources, were presented binaurally and stopped at five simulated distances from the participant’s head. Each sound was presented with five repetitions per distance, resulting in a total of 75 randomised trials (3 sounds × 5 distances × 5 repetitions). This repetition number is consistent with established APA research protocols^65,66^ and provides stable estimates of APA timing while minimizing participant fatigue. Each block of 25 trials was followed by a 2-minute rest interval to minimise fatigue. Prior to the test, a training phase comprising 10 trials (5 distances as in the main tasks × 2 repetitions) with complex tones (100, 450, 1450, 2450 Hz) was conducted to familiarise participants with the response paradigm.

### Task 2: auditory distance estimation

The second task was made to assess the spatial perception of auditory stimuli. The same three sound types and five distances were presented (4 repetitions per condition; 60 trials total) in randomised order. Still blindfolded, participants were instructed to extend their dominant upper arm horizontally as a proprioceptive metric for sound distance, with the shoulder being the most proximal point of reference and the middle fingertip as the most distal point of reference. Then, participants were instructed to approach their nondominant index finger towards the dominant upper arm until it physically reached the perceived stopping position. While this approach deviates from common distance evaluation paradigms (e.g. verbal evaluation^13^), it offered four advantages:

1. it provided a highly ecological, non-conceptual measure of spatial perception that directly couples perception with action^67^, avoiding cognitive mediation inherent in verbal numerical estimates;
2. it aligned with theoretical frameworks emphasizing body-scaled perception^68^, where spatial representations are naturally body-centred and action-based^1^;
3. our stopping distances spanned a narrow range within PPS (0.3–0.7 m); arm-pointing provided a scaled-down analog of blind-walking methods^18^ that enhanced precision for discriminating near-field distances;
4. it was consistent with established practices in near-field auditory distance research^69^.

Finally, experimental blocks, rest intervals, and training procedure were identical to Task 1.

### Task 3: affective evaluation of sounds

The third task assessed the subjective emotional evaluation of the stimuli. The blindfold was removed, and participants used a touchscreen laptop with a screen of 15 inches to provide affective ratings using the Self-Assessment Manikin (SAM) scale^38^. Each 15 stimulus (3 sounds × 5 distances) was rated twice for two affective dimensions: Valence (1 = most negative; 9 = most positive) and Arousal (1 = least arousing; 9 = most arousing). Stimuli were presented in randomised order across all combinations of sound type and distance.

### Task 4: MISS

To gauge individual suggestibility to embodied auditory cues, participants were asked to respond to the Multidimensional Iowa Suggestibility Scale (MISS)^34^, a validated self-report inventory that assesses trait suggestibility across several domains rather than a single context (e.g., hypnosis). Among its five subscales, we focused on Physiological Reactivity (PHR), a 13-item measure of automatic bodily responses to internal or external cues (e.g., “Thinking about something scary can make my heart pound”), because it most closely aligns with our perceptual tasks.

## Data analysis and preparation

### Task 1: premotor reaction time

For each trial, the premotor reaction time (pm-RT) defined the initiation of muscle contraction (i.e., the timing of feedforward motor commands) by measuring the interval between the auditory stimulus offset and the onset of muscle activity in the erector spinae, recorded bilaterally^18,28,41^. The end of the auditory stimulus (trigger offset) was determined from the analogue trigger signal recorded during playback. After DC offset correction (mean of the first 200 samples), the signal was scanned to detect the global peak, followed by the signal descent below a dynamic threshold set at 10% of the peak amplitude. Instead, erector spinae onset was identified from EMG signals first low-pass filtered at 200 Hz (4th-order Butterworth) and then rectified. The envelope was computed via a secondary low-pass filter at 0.02 Hz (5th-order Butterworth). A dynamic threshold for activation was set as the mean baseline activity plus three standard deviations, calculated over a 100-sample window (i.e., 50 ms at 2000 Hz sampling rate) immediately preceding movement onset^18,65^. Trials with pm-RT less than 100 ms were automatically excluded as premature responses, and all remaining trials were visually inspected to remove any further physiologically implausible activations (less than 1% of total trials).

### Task 2: distance estimation

To estimate perceived sound location, we computed the Euclidean 3D distance between the participant’s pointing index finger and the ipsilateral shoulder, based on VICON motion capture data recorded during the final posture of the distance estimation task. The analysis window began 3 s after the sound offset, capturing the response phase. Within this window, a 15-sample moving average filter was applied to reduce measurement noise, and finger stabilisation was identified as periods where the finger’s 3D velocity remained below 0.01 m/s for at least 40 ms^41^. The final distance estimate was calculated as the Euclidean distance between the mean position of the stabilised pointing finger and the dominant-side shoulder.

### Task 3: affective ratings

For each participant, the valence and arousal ratings collected by employing the SAM mannequin were averaged over repetitions, yielding one valence and one arousal score per stimulus type and distance pair.

### Task 4: PHR score

Participants’ raw PHR scores were obtained by summing the 13 items (each rated 1 to 5), which were used as a continuous predictor in the Bayesian models for Tasks 1 and 2.

### Statistical analysis

The statistical inference workflow combined frequentist and Bayesian approaches to evaluate the effects of stimulus type and sound distance on behavioural and physiological responses. The section begins with an analysis of group-level differences using repeated-measures ANOVA. The analysis of differences is followed by Bayesian linear and generalised regression models tailored to the distributional characteristics of each dependent variable, allowing us to quantify uncertainty and test directed hypotheses on the relationships between measured quantities and experimental factors. Then, we explore how suggestibility traits, as captured by the MISS questionnaire, modulate perceptual outcomes.

### Analysis of the differences

Prior to statistical analysis, measurements were averaged over repetitions to guarantee stable estimates of individual performance and reduce the influence of trial-level noise or outliers. When reporting means and standard errors for each stimulus type, we applied the Morey (2008) correction for within-subjects designs^42^, which removes between-subject variability to accurately represent the within-subject effects being tested. This correction was applied only to standard errors of the mean (SEM) for display purposes.

For the measurements of valence and arousal as well as for perceived distance metric, given the lack of normality on the residuals, we run a two-way aligned rank transform (ART) ANOVA^43^ on with the within-subject factors: stimulus type (Applause, Dentist Drill, Pink Noise) and distance (0.3,0.4,0.5,0.6,0.7 m). Distance estimates were z-scored per participant to control for individual differences including arm length (Pearson’s *r* = .46, *p* = .008); valence and arousal ratings were analysed unnormalised. Effect sizes (generalised eta squared, η²) were computed for all significant effects. For significant main effects or interactions, post hoc pairwise comparisons were conducted using sum-to-zero contrasts with Tukey correction for multiple comparisons. Post hoc contrasts were performed using the ART procedure for contrasts (ART-C)^44^. This method allows for valid nonparametric comparisons of factor levels following an ART ANOVA by applying linear contrasts to aligned-and-ranked data, while preserving the structure of factorial designs.

Statistical evaluation for the pm-RT metric was performed following a two-way ANOVA with two within-group factors, with five levels of distance and three levels for stimulus type (as for the previously described metrics). As for perceived distances, the pm-RT estimates were z-scored per participant to control for individual differences even if arm length did not correlate with the metric (Pearson’s *r* = .22, *p* = .21). Sphericity corrections (i.e., Greenhouse-Geisser adjustment) were introduced, and linear model residuals followed the normality assumption (*p* > .05). Effect sizes (generalised eta squared, η²) were computed for all significant effects. Post hoc comparisons were conducted using the estimated marginal means (EMMs) framework to follow up significant main effects and interactions identified in the ANOVA. Pairwise contrasts were applied to the EMMs for both stimulus type and distance, with adjustments for multiple comparisons using the Tukey method. Only contrasts with statistically significant adjusted p-values (*p* ≤ .05) were considered in the interpretation of results.

### Bayesian statistical analysis

With the aim of quantifying the relationships between experimental factors and measurements, we adopted a fully probabilistic modelling approach for inference based on Bayesian statistics^39^. For this analysis, we focused on the relationships between perceived distance, pm-RT, sound type, and the PHR score.

#### Model design

Bayesian models were fitted for each behavioural metric, mirroring the full factorial design used in the ANOVA. This approach allowed for direct comparability between frequentist and Bayesian approaches and ensured that all models captured the key experimental manipulations: stimulus type, distance, and their interaction. Metrics were not normalised per individual to preserve between-subject variance; instead, participant-level random intercepts accounted for individual baselines while estimating population-level effects. Importantly, anatomical differences (i.e., arm length) did not correlate with PHR scores (*r* = .03, *p* = .89), indicating that it did not confound the relationship between sensory suggestibility and outcome measures.

The analysis of perceived distance employed a Bayesian beta regression, which appropriately accounted for the bounded and non-Gaussian nature of this measure^70^. Perceived distances were expressed by pointing on the dominant arm, resulting in values between 0 and 1 m. Modelling both the mean and precision as a function of distance, stimulus type, and their interaction enabled the assessment of how both perceived proximity and response variability changed across conditions.

Instead, a Bayesian linear regression with a Gaussian likelihood described the pm-RT variability across experimental conditions. As with the distance model, this model included the full factorial fixed effects and participant-level random intercepts and modelled the residual standard deviation as a function of the same predictors to account for possible heteroscedasticity.

Importantly, we incorporated the normalised PHR score alongside the existing fixed effects (stimulus type, distance, and their interaction), as well as all two- and three-way interactions involving PHR. This extended structure was applied to both the mean$\:\:\mu\:$ and precision $\:\varphi\:$ (or residual $\:\sigma\:$) components of the models, allowing the assessment of how individual differences in suggestibility influenced not only response magnitude but also response variability. Using the Wilkison notation and defining $\:f(\bullet\:)$ and $\:g(\bullet\:)$ as linking functions (i.e., identity for the Gaussian family, or $\:logit$ and $\:log$ respectively for a Beta regression^70^), the models followed this structure:

$$\:f\left(\mu\:\right)=\:\left(Stim\:*\:Dist\:*\:PHR\right)+\left(1|ID\right),$$

$$\:g\left(\varphi\:\right)=\:\left(Stim\:*\:Dist\:*\:PHR\right)+\left(1|ID\right),$$

incorporating stimulus type ($\:Stim$), distance ($\:Dist$), and normalised PHR score ($\:PHR$) as fixed effects. Participant identifiers $\:ID$ were included as a random intercept.

#### Parameter estimation and model evaluation

Model’s parameters were estimated using four Markov Chain Monte Carlo (MCMC) chains, with 4000 iterations per chain and 2000 warm-up steps to reach convergence. Sampling employed Stan, a probabilistic programming language for Bayesian inference and statistical modelling^71^ and its default sampling scheme, No-U-Turn (NUTS), an adaptive form of Hamiltonian Monte Carlo designed to efficiently explore complex posterior distributions without manual tuning^72^. Default weakly informative priors (normal distribution, mean = 0, SD = 1). Convergence diagnostics (R̂ < 1.01) and effective sample sizes were verified for all parameters^39^.

Model adequacy was evaluated using posterior predictive checks, drawing between 2000 samples from the fitted posterior distributions to compare model predictions with observed data across key experimental conditions. We compared the model’s average predictions and how much they varied from the real data. This approach enables the possibility to assess if the model reproduced the patterns observed in the data by visually inspecting summary plots and by employing aggregated statistics.

#### Probabilistic queries

Experimental effects were assessed through probabilistic queries of the posterior distributions of fixed effects. Directional and comparative hypotheses were tested with posterior probabilities for targeted hypotheses (e.g., whether the effect of stimulus Pink Noise was reliably smaller than Dentist Drill in proximity estimation). Posterior distributions were summarised using means and 95% credible intervals (95%-CI), giving a clear range of plausible effect values^39^. To further quantify certainty regarding the direction of these effects, we computed the probability of direction, representing the proportion of posterior samples consistent with the sign of the posterior mean. These metrics allowed us to interpret effect magnitudes and robustness without relying on binary significance thresholds.

To facilitate meaningful comparison between models using different likelihood families (i.e., Gaussian versus Beta regression), we standardised measures of variability. Specifically, for the beta regression, we computed the posterior standard deviation from the precision parameter to allow consistent interpretation of variability across different distributional families^70^.

We computed marginal effects to quantify how changes in predictors influenced outcome variables across their posterior distributions. For continuous predictors (e.g., distance), we estimated average marginal slopes, reflecting the instantaneous rate of change in the outcome per unit increase in the predictor. For categorical predictors (e.g., stimulus type), we computed average contrasts between categories, summarising differences in predicted outcomes across experimental conditions. Marginal effect estimates were summarised as posterior means and 95%-CI, providing robust population-level inferences integrated over observed covariate distributions.

Finally, we conducted post hoc exploratory analyses on model-derived parameters not directly estimated within the primary Bayesian models. Specifically, we extracted participant-level model estimates or their dispersion from posterior draws and then analysed their relationship with individual-level covariates (e.g., standardised PHR scores). For this procedure, we sampled 4000 draws and summarised them with mean and standard deviation over levels. These analyses allowed us to explore whether participant differences in suggestibility explained systematic variation in model uncertainty or sensitivity to experimental manipulations.

## Software tools

Data acquisition and preprocessing were conducted using custom-written scripts in MATLAB. All subsequent statistical analyses were performed in R (version 4.4.2). For data manipulation and visualisation, we used packages including *data.table*, *ggplot2*, *car*, and *Rmisc*. Frequentist analyses (e.g., repeated-measures ANOVA, post hoc contrasts, and effect size estimation) were implemented using *afex*, *ARTool*, *emmeans*, and *effectsize*. Bayesian analyses relied on Stan via the interfaces provided by *brms* and *rstan*, complemented by *marginaleffects*, *tidybayes*, and posterior for marginal effects estimation, posterior exploration, model checking, and hypothesis evaluation.

## Supplementary Information

Below is the link to the electronic supplementary material.

Supplementary Material 1

## Data Availability

The datasets for the current study, as well as the analysis scripts, are available from the corresponding author on request.

## References

[1] Rizzolatti, G., Fadiga, L., Fogassi, L. & Gallese, V. Space around us. *Science* **277**, 190–191 (1997). 10.1126/science.277.5323.190 9235632

[2] Graziano, M. S. A. & Cooke, D. F. Parieto-frontal interactions, personal space, and defensive behavior. *Neuropsychologia* **44**, 2621–2635 (2006). 17128446 10.1016/j.neuropsychologia.2005.09.011

[3] Serino, A., Annella, L. & Avenanti, A. Motor properties of peripersonal space in humans. *PLOS ONE* **4**, e6582 (2009). 19668366 10.1371/journal.pone.0006582 PMC2719059

[4] Ferri, F., Tajadura-Jiménez, A., Väljamäe, A., Vastano, R. & Costantini, M. Emotion-inducing approaching sounds shape the boundaries of multisensory peripersonal space. *Neuropsychologia* **70**, 468–475 (2015). 25744869 10.1016/j.neuropsychologia.2015.03.001

[5] Sambo, C. F. & Iannetti, G. D. Better safe than sorry? The safety margin surrounding the body is increased by anxiety. *J. Neurosci.* **33**, 14225–14230 (2013). 23986256 10.1523/JNEUROSCI.0706-13.2013 PMC6618504

[6] Ardizzi, M. & Ferri, F. Interoceptive influences on peripersonal space boundary. *Cognition* **177**, 79–86 (2018). 29655026 10.1016/j.cognition.2018.04.001

[7] Serino, A. Peripersonal space (PPS) as a multisensory interface between the individual and the environment, defining the space of the self. *Neurosci. Biobehav. Rev.* **99**, 138–159 (2019). 30685486 10.1016/j.neubiorev.2019.01.016

[8] Rizzolatti, G., Scandolara, C., Matelli, M. & Gentilucci, M. Afferent properties of periarcuate neurons in macaque monkeys. II. Visual responses. *Behav. Brain. Res.* **2**, 147–163 (1981). 7248055 10.1016/0166-4328(81)90053-x

[9] Graziano, M. S. A. & Gross, C. G. A bimodal map of space: Somatosensory receptive fields in the macaque putamen with corresponding visual receptive fields. *Exp. Brain Res.* **97**, 96–109 (1993). 8131835 10.1007/BF00228820

[10] Bach, D. R. et al. Rising sound intensity: An intrinsic warning cue activating the amygdala. *Cereb. Cortex*. **18**, 145–150 (2008). 17490992 10.1093/cercor/bhm040

[11] Makin, T. R., Holmes, N. P., Brozzoli, C., Rossetti, Y. & Farnè, A. Coding of visual space during motor preparation: Approaching objects rapidly modulate corticospinal excitability in hand-centered coordinates. *J. Neurosci.* **29**, 11841–11851 (2009). 19776270 10.1523/JNEUROSCI.2955-09.2009 PMC6666640

[12] Finisguerra, A., Canzoneri, E., Serino, A., Pozzo, T. & Bassolino, M. Moving sounds within the peripersonal space modulate the motor system. *Neuropsychologia* **70**, 421–428 (2015). 25281311 10.1016/j.neuropsychologia.2014.09.043

[13] Zahorik, P., Brungart, D. S. & Bronkhorst, A. W. Auditory distance perception in humans: A summary of past and present research. *Acta Acust. United Acust.* **91**, 409–420 (2005).

[14] Bach, D. R., Neuhoff, J. G., Perrig, W. & Seifritz, E. Looming sounds as warning signals: The function of motion cues. *Int. J. Psychophysiol.* **74**, 28–33 (2009). 19615414 10.1016/j.ijpsycho.2009.06.004

[15] Seifritz, E. et al. Neural processing of auditory looming in the human brain. *Curr. Biol.* **12**, 2147–2151 (2002). 12498691 10.1016/s0960-9822(02)01356-8

[16] McDonald, J. J., Teder-Sälejärvi, W. A. & Hillyard, S. A. Involuntary orienting to sound improves visual perception. *Nature* **407**, 906–908 (2000). 11057669 10.1038/35038085

[17] Canzoneri, E., Magosso, E. & Serino, A. Dynamic sounds capture the boundaries of peripersonal space representation in humans. *PLOS ONE*. **7**, e44306 (2012). 23028516 10.1371/journal.pone.0044306 PMC3460958

[18] Camponogara, I., Komeilipoor, N. & Cesari, P. When distance matters: Perceptual bias and behavioral response for approaching sounds in peripersonal and extrapersonal space. *Neuroscience* **304**, 101–108 (2015). 26208838 10.1016/j.neuroscience.2015.07.054

[19] van der Heijden, K., Rauschecker, J. P., de Gelder, B. & Formisano, E. Cortical mechanisms of spatial hearing. *Nat. Rev. Neurosci.* **20**, 609–623 (2019). 31467450 10.1038/s41583-019-0206-5 PMC7081609

[20] Ahveninen, J. et al. Task-modulated what and where pathways in human auditory cortex. *Proc. Natl. Acad. Sci.* **103**, 14608–14613 (2006). 16983092 10.1073/pnas.0510480103 PMC1600007

[21] Grantham, D. W. Auditory motion perception: Snapshots revisited. In *Binaural and Spatial Hearing in Real and Virtual Environments*. 295–313 (Lawrence Erlbaum, 1997).

[22] Roggerone, V., Vacher, J., Tarlao, C. & Guastavino, C. Auditory motion perception emerges from successive sound localizations integrated over time. *Sci. Rep.* **9**, 16437 (2019). 31712688 10.1038/s41598-019-52742-0 PMC6848124

[23] Aruin, A. & Latash, M. The role of motor action in anticipatory postural adjustments studied with self-induced and externally triggered perturbations. *Exp. Brain Res.* **106**, 291-300 (1995). 10.1007/BF00241125 8566194

[24] Massion, J. Movement, posture and equilibrium: Interaction and coordination. *Prog. Neurobiol.* **38**, 35–56 (1992). 1736324 10.1016/0301-0082(92)90034-c

[25] Cesari, P., Piscitelli, F., Pascucci, F. & Bertucco, M. Postural threat influences the coupling between anticipatory and compensatory postural adjustments in response to an external perturbation. *Neuroscience* **490**, 25–35 (2022). 35276303 10.1016/j.neuroscience.2022.03.005

[26] Maffei, G., Herreros, I., Sanchez-Fibla, M., Friston, K. J. & Verschure, P. F. M. J. The perceptual shaping of anticipatory actions. *Proc. R. Soc. B Biol. Sci.* **284**, 20171780 (2017). 10.1098/rspb.2017.1780 PMC5745402 29263282

[27] Bouisset, S. & Zattara, M. Biomechanical study of the programming of anticipatory postural adjustments associated with voluntary movement. *J. Biomech.* **20**, 735–742 (1987). 3654672 10.1016/0021-9290(87)90052-2

[28] Bahadori, M., Barumerli, R., Geronazzo, M. & Cesari, P. Action planning and affective states within the auditory peripersonal space in normal hearing and cochlear-implanted listeners. *Neuropsychologia* **155**, 107790 (2021). 33636155 10.1016/j.neuropsychologia.2021.107790

[29] Noel, J. P., Blanke, O. & Serino, A. From multisensory integration in peripersonal space to bodily self-consciousness: From statistical regularities to statistical inference. *Ann. N. Y. Acad. Sci.* **1426**, 146–165 (2018). 10.1111/nyas.13867 29876922

[30] Marotta, A., Tinazzi, M., Cavedini, C., Zampini, M. & Fiorio, M. Individual differences in the rubber hand illusion are related to sensory suggestibility. *PLoS ONE*. **11**, e0168489 (2016). 27977783 10.1371/journal.pone.0168489 PMC5158054

[31] Walsh, E. et al. Are you suggesting that’s my hand? The relation between hypnotic suggestibility and the rubber hand illusion. *Perception* **44**, 709–723 (2015). 26489211 10.1177/0301006615594266

[32] Biggio, M. et al. Surrounded, detached: the relationship between defensive peripersonal space and personality. *Front. Psychiatry* **14** (2023). 10.3389/fpsyt.2023.1244364 PMC10603239 37900289

[33] De Vignemont, F. & Iannetti, G. D. How many peripersonal spaces? *Neuropsychologia* **70**, 327–334 (2015). 10.1016/j.neuropsychologia.2014.11.018 25448854

[34] Kotov, R. I., Bellman, S. B. & Watson, D. B. *Multidimensional Iowa Suggestibility Scale (MISS) Brief Manual* (2004).

[35] Kolarik, A. J., Moore, B. C. J., Zahorik, P., Cirstea, S. & Pardhan, S. Auditory distance perception in humans: A review of cues, development, neuronal bases, and effects of sensory loss. *Attent. Percept. Psychophys.* **78**, 373–395 (2016). 10.3758/s13414-015-1015-1 PMC4744263 26590050

[36] Gladwin, T. E., Hashemi, M. M., van Ast, V. & Roelofs, K. Ready and waiting: Freezing as active action preparation under threat. *Neurosci. Lett.* **619**, 182–188 (2016). 26994781 10.1016/j.neulet.2016.03.027

[37] Bradley, M. M. & Lang, P. J. *The International Affective Digitized Sounds**(IADS-2): Affective Ratings of Sounds and Instruction Manual* (2007).

[38] Bradley, M. M. & Lang, P. J. Measuring emotion: The self-assessment manikin and the semantic differential. *J. Behav. Ther. Exp. Psychiatry*. **25**, 49–59 (1994). 7962581 10.1016/0005-7916(94)90063-9

[39] Gelman, A. et al. *Bayesian Data Analysis*. 3rd Ed. (Chapman Hall/CRC, 2013).

[40] Neuhoff, J. G. An adaptive bias in the perception of looming auditory motion. *Ecol. Psychol.* **13**, 87–110 (2001).

[41] Geronazzo, M., Barumerli, R. & Cesari, P. Shaping the auditory peripersonal space with motor planning in immersive virtual reality. *Virtual Real. *3067–3087 10.1007/s10055-023-00854-4 (2023).

[42] Morey, R. D. Confidence intervals from normalized data: A correction to Cousineau (2005). *TQMP* **4**, 61–64 (2008).

[43] Wobbrock, J. O., Findlater, L., Gergle, D. & Higgins, J. J. The aligned rank transform for nonparametric factorial analyses using only anova procedures. In *Proceedings of the SIGCHI Conference on Human Factors in Computing Systems*. 143–146. 10.1145/1978942.1978963 (ACM, 2011).

[44] Elkin, L. A., Kay, M., Higgins, J. J. & Wobbrock, J. O. An aligned rank transform procedure for multifactor contrast tests. In *The 34th Annual ACM Symposium on User Interface Software and Technology*. 754–768. 10.1145/3472749.3474784 (ACM, 2021).

[45] Middlebrooks, J. C. Sound localization. In *Handbook of Clinical Neurology*. Vol. 129. 99–116 (Elsevier, 2015). 10.1016/B978-0-444-62630-1.00006-8 25726265

[46] Neuhoff, J. G. Perceptual bias for rising tones. *Nature* **395**, 123–124 (1998). 9744266 10.1038/25862

[47] Bizley, J. K. & Cohen, Y. E. The what, where and how of auditory-object perception. *Nat. Rev. Neurosci.* **14**, 693–707 (2013). 24052177 10.1038/nrn3565 PMC4082027

[48] Pannese, A., Grandjean, D. & Frühholz, S. Amygdala and auditory cortex exhibit distinct sensitivity to relevant acoustic features of auditory emotions. *Cortex* **85**, 116–125 (2016). 27855282 10.1016/j.cortex.2016.10.013

[49] Ignatiadis, K. et al. Cortical signatures of auditory looming bias show cue-specific adaptation between newborns and young adults. *Commun. Psychol.* **2**, 1–15 (2024). 38859821 10.1038/s44271-024-00105-5 PMC11163589

[50] Yang, W. et al. Affective auditory stimulus database: An expanded version of the International Affective Digitized Sounds (IADS-E). *Behav. Res.* **50**, 1415–1429 (2018). 10.3758/s13428-018-1027-6 29520632

[51] Ignatiadis, K., Barumerli, R., Deco, G., Tóth, B. & Baumgartner, R. Threat-related corticocortical connectivity elicited by rapid auditory looms. *Sci. Rep.* **16**, 834 (2025). 41387490 10.1038/s41598-025-30552-x PMC12780023

[52] de Vignemont, F. & Farnè, A. Peripersonal space: Why so last-second? *Philosophical Trans. Royal Soc. B: Biol. Sci.* **379**, 20230159 (2024). 10.1098/rstb.2023.0159 PMC11529623 39155714

[53] Rauschecker, J. P. Where, when, and how: Are they all sensorimotor? Towards a unified view of the dorsal pathway in vision and audition. *Cortex* **98**, 262–268 (2018). 29183630 10.1016/j.cortex.2017.10.020 PMC5771843

[54] Koban, L., Jepma, M., Geuter, S. & Wager, T. D. What’s in a word? How instructions, suggestions, and social information change pain and emotion. *Neuroscience Biobehavioral Reviews*. **81**, 29–42 (2017). 29173508 10.1016/j.neubiorev.2017.02.014 PMC5706563

[55] Rossi Sebastiano, A. et al. Multisensory-driven facilitation within the peripersonal space is modulated by the expectations about stimulus location on the body. *Sci. Rep.* **12**, 20061 (2022). 36414633 10.1038/s41598-022-21469-w PMC9681840

[56] Matsuda, Y., Sugimoto, M., Inami, M. & Kitazaki, M. Peripersonal space in the front, rear, left and right directions for audio-tactile multisensory integration. *Sci. Rep.* **11**, 11303 (2021). 34050213 10.1038/s41598-021-90784-5 PMC8163804

[57] Beatty, G. F., Cranley, N. M., Carnaby, G. & Janelle, C. M. Emotions predictably modify response times in the initiation of human motor actions: A meta-analytic review. *Emotion* **16**, 237–251 (2016). 26461243 10.1037/emo0000115

[58] Lu, J., Kemmerer, S. K., Riecke, L. & de Gelder, B. Early threat perception is independent of later cognitive and behavioral control. A virtual reality-EEG-ECG study. *Cereb. Cortex*. **33**, 8748–8758 (2023). 37197766 10.1093/cercor/bhad156 PMC10321083

[59] Baumgartner, R. et al. Asymmetries in behavioral and neural responses to spectral cues demonstrate the generality of auditory looming bias. *Proc. Natl. Acad. Sci. USA*. **114**, 9743–9748 (2017). 28827336 10.1073/pnas.1703247114 PMC5594652

[60] Jicol, C. et al. Imagine That! Imaginative suggestibility affects presence in virtual reality. In *Proceedings of the CHI Conference on Human Factors in Computing Systems*. 1–11. 10.1145/3544548.3581212 (ACM, 2023).

[61] Lebert, A., Chaby, L., Garnot, C. & Vergilino-Perez, D. The impact of emotional videos and emotional static faces on postural control through a personality trait approach. *Exp. Brain Res.* **238**, 2877–2886 (2020). 33057868 10.1007/s00221-020-05941-5

[62] Bahadori, M. & Cesari, P. Affective sounds entering the peripersonal space influence the whole-body action preparation. *Neuropsychologia* **159**, 107917 (2021). 34153305 10.1016/j.neuropsychologia.2021.107917

[63] Greif, T., Barumerli, R., Ignatiadis, K., Tóth, B. & Baumgartner, R. The role of spatial perception in auditory looming bias: Neurobehavioral evidence from impossible ears. *Front. Neurosci.* **19** (2025). 10.3389/fnins.2025.1645936 PMC12414998 40927421

[64] De Raad, B. *The Big Five Personality Factors: The Psycholexical Approach to Personality*. Vol. VII. 128 (Hogrefe & Huber Publishers, 2000).

[65] Bertucco, M. & Cesari, P. Does movement planning follow Fitts’ law? Scaling anticipatory postural adjustments with movement speed and accuracy. *Neuroscience* **171**, 205–213 (2010). 20804822 10.1016/j.neuroscience.2010.08.023

[66] Liang, H., Kaewmanee, T. & Aruin, A. S. The role of an auditory cue in generating anticipatory postural adjustments in response to an external perturbation. *Exp. Brain Res.* **238**, 631–641 (2020). 32009192 10.1007/s00221-020-05738-6

[67] Cesari, P. & Newell, K. M. The scaling of human grip configurations. *J. Exp. Psychol. Hum. Percept. Perform.* **25**, 927–935 (1999). 10464939 10.1037//0096-1523.25.4.927

[68] Warren, W. H. Perceiving affordances: Visual guidance of stair climbing. *J. Exp. Psychol. Hum. Percept. Perform.* **10**, 683–703 (1984). 6238127 10.1037//0096-1523.10.5.683

[69] Parseihian, G., Jouffrais, C. & Katz, B. F. G. Reaching nearby sources: Comparison between real and virtual sound and visual targets. *Front. Neurosci*. **8**, 1-13 (2014). 10.3389/fnins.2014.00269 PMC4151089 25228855

[70] Cribari-Neto, F. & Zeileis, A. Beta regression in R. *J Stat. Soft* **34**, 1-24 (2010).

[71] Carpenter, B. et al. Stan: A probabilistic programming language. *J. Stat. Softw.* **76**, 1–32 (2017). 36568334 10.18637/jss.v076.i01 PMC9788645

[72] Hoffman, M. D. & Gelman, A. The no-U-turn sampler: Adaptively setting path lengths in Hamiltonian Monte Carlo. *J. Mach. Learn. Res.* **15**, 1593–1623 (2014).

