# Supplementary material for: Semantic processing and individual suggestibility modulate motor preparation and perceived distance for looming sounds entering the peripersonal space
Source: Sci Rep. 2026 Apr 20;16:18158. doi: 10.1038/s41598-026-48067-4 (PMC13254331; doi:10.1038/s41598-026-48067-4)
Supplement: Supplementary file 1 — Supplementary Material 1 [file 41598_2026_48067_MOESM1_ESM.pdf]

Supplementary materials

Title: Semantic processing and individual suggestibility modulate motor preparation and perceived distance for looming sounds entering the peripersonal space.

Roberto Barumerli, Michele Geronazzo, Paola Cesari

Contact: roberto.barumerli@univr.it

These supplementary materials report post-hoc analyses for perceived distance and premotor reaction time (pm-RT), based on within-participant z-scored measures. Full details of the statistical approach are reported in the Results and Methods sections of the main manuscript.

Table of contents

1. Normalised perceived distance 2
   1.1. Table S1: Distance contrasts 2
   1.2. Table S2: Stimulus contrasts 2
   1.3. Table S3: Interaction between Distance and Stimulus 2
2. Normalised Pre-Motor Reaction Time 7
   2.1. Table S4: Distance contrasts 7
   2.2. Table S5: Stimulus contrasts 7
   2.3. Table S6: Distance contrasts BY Stimulus 7
   2.4. Table S7: Stimulus contrasts BY Distance 8

1. Normalised perceived distance

1.1. Table S1: Distance contrasts

contrast	Diff (a.u.)	SE	Diff (rank)	SE (rank)	df	t.ratio	p.value
0.3 - 0.4	-0.744	0.049	-94.626	10.087	480	-9.38	**<0.001**
0.3 - 0.5	-1.283	0.046	-181.859	10.087	480	-18.03	**<0.001**
0.3 - 0.6	-1.845	0.052	-278.394	10.087	480	-27.60	**<0.001**
0.3 - 0.7	-2.275	0.050	-348.152	10.087	480	-34.52	**<0.001**
0.4 - 0.5	-0.538	0.042	-87.232	10.087	480	-8.65	**<0.001**
0.4 - 0.6	-1.101	0.049	-183.768	10.087	480	-18.22	**<0.001**
0.4 - 0.7	-1.531	0.046	-253.525	10.087	480	-25.13	**<0.001**
0.5 - 0.6	-0.563	0.046	-96.535	10.087	480	-9.57	**<0.001**
0.5 - 0.7	-0.993	0.043	-166.293	10.087	480	-16.49	**<0.001**
0.6 - 0.7	-0.430	0.050	-69.758	10.087	480	-6.92	**<0.001**

1.2. Table S2: Stimulus contrasts

contrast	Diff (a.u.)	SE	Diff (rank)	SE (rank)	df	t.ratio	p.value
PinkNoise - Applause	-0.578	0.073	-167.279	13.592	480	-12.31	**<0.001**
PinkNoise - DentistDrill	-0.529	0.081	-148.158	13.592	480	-10.90	**<0.001**
Applause - DentistDrill	0.049	0.077	19.121	13.592	480	1.41	0.338

1.3. Table S3: Interaction between Distance and Stimulus

contrast	Diff (a.u.)	SE	Diff (rank)	SE (rank)	df	t.ratio	p.value
Applause,0.3 - Applause,0.4	-1.006	0.115	-137.424	16.795	480	-8.18	**<0.001**
Applause,0.3 - Applause,0.5	-1.527	0.122	-222.545	16.795	480	-13.25	**<0.001**
Applause,0.3 - Applause,0.6	-1.832	0.110	-276.212	16.795	480	-16.45	**<0.001**
Applause,0.3 - Applause,0.7	-2.371	0.130	-347.636	16.795	480	-20.70	**<0.001**
Applause,0.3 - DentistDrill,0.3	-0.058	0.138	-9.697	16.795	480	-0.58	1.000
Applause,0.3 - DentistDrill,0.4	-0.719	0.125	-96.545	16.795	480	-5.75	**<0.001**
Applause,0.3 - DentistDrill,0.5	-1.239	0.113	-175.636	16.795	480	-10.46	**<0.001**
Applause,0.3 - DentistDrill,0.6	-2.072	0.116	-309.606	16.795	480	-18.43	**<0.001**
Applause,0.3 - DentistDrill,0.7	-2.400	0.106	-356.212	16.795	480	-21.21	**<0.001**

contrast	Diff (a.u.)	SE	Diff (rank)	SE (rank)	df	t.ratio	p.value
Applause,0.3 - PinkNoise,0.3	0.333	0.104	39.364	16.795	480	2.34	0.558
Applause,0.3 - PinkNoise,0.4	-0.233	0.107	-26.939	16.795	480	-1.60	0.957
Applause,0.3 - PinkNoise,0.5	-0.807	0.115	-108.455	16.795	480	-6.46	**<0.001**
Applause,0.3 - PinkNoise,0.6	-1.358	0.129	-194.091	16.795	480	-11.56	**<0.001**
Applause,0.3 - PinkNoise,0.7	-1.781	0.112	-266.545	16.795	480	-15.87	**<0.001**
Applause,0.4 - Applause,0.5	-0.521	0.116	-85.121	16.795	480	-5.07	**<0.001**
Applause,0.4 - Applause,0.6	-0.826	0.103	-138.788	16.795	480	-8.26	**<0.001**
Applause,0.4 - Applause,0.7	-1.365	0.124	-210.212	16.795	480	-12.52	**<0.001**
Applause,0.4 - DentistDrill,0.3	0.948	0.132	127.727	16.795	480	7.60	**<0.001**
Applause,0.4 - DentistDrill,0.4	0.287	0.119	40.879	16.795	480	2.43	0.491
Applause,0.4 - DentistDrill,0.5	-0.233	0.106	-38.212	16.795	480	-2.28	0.610
Applause,0.4 - DentistDrill,0.6	-1.066	0.110	-172.182	16.795	480	-10.25	**<0.001**
Applause,0.4 - DentistDrill,0.7	-1.394	0.099	-218.788	16.795	480	-13.03	**<0.001**
Applause,0.4 - PinkNoise,0.3	1.339	0.096	176.788	16.795	480	10.53	**<0.001**
Applause,0.4 - PinkNoise,0.4	0.773	0.100	110.485	16.795	480	6.58	**<0.001**
Applause,0.4 - PinkNoise,0.5	0.199	0.108	28.970	16.795	480	1.72	0.925
Applause,0.4 - PinkNoise,0.6	-0.352	0.123	-56.667	16.795	480	-3.37	0.056
Applause,0.4 - PinkNoise,0.7	-0.775	0.105	-129.121	16.795	480	-7.69	**<0.001**
Applause,0.5 - Applause,0.6	-0.305	0.112	-53.667	16.795	480	-3.20	0.094
Applause,0.5 - Applause,0.7	-0.844	0.131	-125.091	16.795	480	-7.45	**<0.001**
Applause,0.5 - DentistDrill,0.3	1.469	0.139	212.848	16.795	480	12.67	**<0.001**
Applause,0.5 - DentistDrill,0.4	0.808	0.126	126.000	16.795	480	7.50	**<0.001**
Applause,0.5 - DentistDrill,0.5	0.287	0.114	46.909	16.795	480	2.79	0.254
Applause,0.5 - DentistDrill,0.6	-0.545	0.117	-87.061	16.795	480	-5.18	**<0.001**
Applause,0.5 - DentistDrill,0.7	-0.873	0.107	-133.667	16.795	480	-7.96	**<0.001**
Applause,0.5 - PinkNoise,0.3	1.860	0.105	261.909	16.795	480	15.59	**<0.001**
Applause,0.5 - PinkNoise,0.4	1.294	0.109	195.606	16.795	480	11.65	**<0.001**
Applause,0.5 - PinkNoise,0.5	0.720	0.116	114.091	16.795	480	6.79	**<0.001**

contrast	Diff (a.u.)	SE	Diff (rank)	SE (rank)	df	t.ratio	p.value
Applause,0.5 - PinkNoise,0.6	0.169	0.130	28.455	16.795	480	1.69	0.934
Applause,0.5 - PinkNoise,0.7	-0.254	0.113	-44.000	16.795	480	-2.62	0.359
Applause,0.6 - Applause,0.7	-0.539	0.120	-71.424	16.795	480	-4.25	**0.002**
Applause,0.6 - DentistDrill,0.3	1.774	0.129	266.515	16.795	480	15.87	**<0.001**
Applause,0.6 - DentistDrill,0.4	1.113	0.115	179.667	16.795	480	10.70	**<0.001**
Applause,0.6 - DentistDrill,0.5	0.592	0.102	100.576	16.795	480	5.99	**<0.001**
Applause,0.6 - DentistDrill,0.6	-0.240	0.105	-33.394	16.795	480	-1.99	0.805
Applause,0.6 - DentistDrill,0.7	-0.568	0.094	-80.000	16.795	480	-4.76	**<0.001**
Applause,0.6 - PinkNoise,0.3	2.165	0.091	315.576	16.795	480	18.79	**<0.001**
Applause,0.6 - PinkNoise,0.4	1.599	0.095	249.273	16.795	480	14.84	**<0.001**
Applause,0.6 - PinkNoise,0.5	1.025	0.104	167.758	16.795	480	9.99	**<0.001**
Applause,0.6 - PinkNoise,0.6	0.474	0.119	82.121	16.795	480	4.89	**<0.001**
Applause,0.6 - PinkNoise,0.7	0.051	0.101	9.667	16.795	480	0.58	1.000
Applause,0.7 - DentistDrill,0.3	2.313	0.146	337.939	16.795	480	20.12	**<0.001**
Applause,0.7 - DentistDrill,0.4	1.652	0.134	251.091	16.795	480	14.95	**<0.001**
Applause,0.7 - DentistDrill,0.5	1.132	0.122	172.000	16.795	480	10.24	**<0.001**
Applause,0.7 - DentistDrill,0.6	0.299	0.126	38.030	16.795	480	2.26	0.618
Applause,0.7 - DentistDrill,0.7	-0.029	0.116	-8.576	16.795	480	-0.51	1.000
Applause,0.7 - PinkNoise,0.3	2.704	0.114	387.000	16.795	480	23.04	**<0.001**
Applause,0.7 - PinkNoise,0.4	2.138	0.117	320.697	16.795	480	19.09	**<0.001**
Applause,0.7 - PinkNoise,0.5	1.564	0.124	239.182	16.795	480	14.24	**<0.001**
Applause,0.7 - PinkNoise,0.6	1.013	0.137	153.545	16.795	480	9.14	**<0.001**
Applause,0.7 - PinkNoise,0.7	0.590	0.122	81.091	16.795	480	4.83	**<0.001**
DentistDrill,0.3 - DentistDrill,0.4	-0.661	0.142	-86.848	16.795	480	-5.17	**<0.001**
DentistDrill,0.3 - DentistDrill,0.5	-1.181	0.131	-165.939	16.795	480	-9.88	**<0.001**
DentistDrill,0.3 - DentistDrill,0.6	-2.014	0.134	-299.909	16.795	480	-17.86	**<0.001**
DentistDrill,0.3 - DentistDrill,0.7	-2.342	0.125	-346.515	16.795	480	-20.63	**<0.001**
DentistDrill,0.3 - PinkNoise,0.3	0.391	0.123	49.061	16.795	480	2.92	0.190

contrast	Diff (a.u.)	SE	Diff (rank)	SE (rank)	df	t.ratio	p.value
DentistDrill,0.3 - PinkNoise,0.4	-0.175	0.126	-17.242	16.795	480	-1.03	0.999
DentistDrill,0.3 - PinkNoise,0.5	-0.749	0.132	-98.758	16.795	480	-5.88	**<0.001**
DentistDrill,0.3 - PinkNoise,0.6	-1.300	0.145	-184.394	16.795	480	-10.98	**<0.001**
DentistDrill,0.3 - PinkNoise,0.7	-1.722	0.130	-256.848	16.795	480	-15.29	**<0.001**
DentistDrill,0.4 - DentistDrill,0.5	-0.520	0.118	-79.091	16.795	480	-4.71	**<0.001**
DentistDrill,0.4 - DentistDrill,0.6	-1.353	0.121	-213.061	16.795	480	-12.69	**<0.001**
DentistDrill,0.4 - DentistDrill,0.7	-1.681	0.111	-259.667	16.795	480	-15.46	**<0.001**
DentistDrill,0.4 - PinkNoise,0.3	1.052	0.109	135.909	16.795	480	8.09	**<0.001**
DentistDrill,0.4 - PinkNoise,0.4	0.487	0.112	69.606	16.795	480	4.14	**0.004**
DentistDrill,0.4 - PinkNoise,0.5	-0.088	0.119	-11.909	16.795	480	-0.71	1.000
DentistDrill,0.4 - PinkNoise,0.6	-0.639	0.133	-97.545	16.795	480	-5.81	**<0.001**
DentistDrill,0.4 - PinkNoise,0.7	-1.061	0.117	-170.000	16.795	480	-10.12	**<0.001**
DentistDrill,0.5 - DentistDrill,0.6	-0.833	0.108	-133.970	16.795	480	-7.98	**<0.001**
DentistDrill,0.5 - DentistDrill,0.7	-1.161	0.097	-180.576	16.795	480	-10.75	**<0.001**
DentistDrill,0.5 - PinkNoise,0.3	1.572	0.094	215.000	16.795	480	12.80	**<0.001**
DentistDrill,0.5 - PinkNoise,0.4	1.007	0.098	148.697	16.795	480	8.85	**<0.001**
DentistDrill,0.5 - PinkNoise,0.5	0.432	0.106	67.182	16.795	480	4.00	**0.006**
DentistDrill,0.5 - PinkNoise,0.6	-0.119	0.121	-18.455	16.795	480	-1.10	0.999
DentistDrill,0.5 - PinkNoise,0.7	-0.541	0.103	-90.909	16.795	480	-5.41	**<0.001**
DentistDrill,0.6 - DentistDrill,0.7	-0.328	0.101	-46.606	16.795	480	-2.77	0.264
DentistDrill,0.6 - PinkNoise,0.3	2.405	0.098	348.970	16.795	480	20.78	**<0.001**
DentistDrill,0.6 - PinkNoise,0.4	1.839	0.102	282.667	16.795	480	16.83	**<0.001**
DentistDrill,0.6 - PinkNoise,0.5	1.265	0.110	201.152	16.795	480	11.98	**<0.001**
DentistDrill,0.6 - PinkNoise,0.6	0.714	0.125	115.515	16.795	480	6.88	**<0.001**
DentistDrill,0.6 - PinkNoise,0.7	0.291	0.107	43.061	16.795	480	2.56	0.397
DentistDrill,0.7 - PinkNoise,0.3	2.733	0.086	395.576	16.795	480	23.55	**<0.001**
DentistDrill,0.7 - PinkNoise,0.4	2.167	0.091	329.273	16.795	480	19.61	**<0.001**
DentistDrill,0.7 - PinkNoise,0.5	1.593	0.099	247.758	16.795	480	14.75	**<0.001**

contrast	Diff (a.u.)	SE	Diff (rank)	SE (rank)	df	t.ratio	p.value
DentistDrill,0.7 - PinkNoise,0.6	1.042	0.115	162.121	16.795	480	9.65	**<0.001**
DentistDrill,0.7 - PinkNoise,0.7	0.619	0.096	89.667	16.795	480	5.34	**<0.001**
PinkNoise,0.3 - PinkNoise,0.4	-0.566	0.087	-66.303	16.795	480	-3.95	**0.008**
PinkNoise,0.3 - PinkNoise,0.5	-1.140	0.096	-147.818	16.795	480	-8.80	**<0.001**
PinkNoise,0.3 - PinkNoise,0.6	-1.691	0.113	-233.455	16.795	480	-13.90	**<0.001**
PinkNoise,0.3 - PinkNoise,0.7	-2.114	0.093	-305.909	16.795	480	-18.21	**<0.001**
PinkNoise,0.4 - PinkNoise,0.5	-0.574	0.100	-81.515	16.795	480	-4.85	**<0.001**
PinkNoise,0.4 - PinkNoise,0.6	-1.125	0.116	-167.152	16.795	480	-9.95	**<0.001**
PinkNoise,0.4 - PinkNoise,0.7	-1.548	0.097	-239.606	16.795	480	-14.27	**<0.001**
PinkNoise,0.5 - PinkNoise,0.6	-0.551	0.123	-85.636	16.795	480	-5.10	**<0.001**
PinkNoise,0.5 - PinkNoise,0.7	-0.974	0.105	-158.091	16.795	480	-9.41	**<0.001**
PinkNoise,0.6 - PinkNoise,0.7	-0.423	0.121	-72.455	16.795	480	-4.31	**0.002**

2. Normalised Pre-Motor Reaction Time

2.1. Table S4: Distance contrasts

contrast	Estimate (a.u.)	SE	df	t.ratio	p.value
0.6 - 0.7	-0.456	0.124	32	-3.68	**0.007**
0.6 - 0.5	0.270	0.117	32	2.30	0.172
0.6 - 0.4	0.485	0.136	32	3.57	**0.009**
0.6 - 0.3	0.700	0.127	32	5.51	**<0.001**
0.7 - 0.5	0.726	0.070	32	10.37	**<0.001**
0.7 - 0.4	0.941	0.120	32	7.85	**<0.001**
0.7 - 0.3	1.156	0.126	32	9.14	**<0.001**
0.5 - 0.4	0.215	0.112	32	1.92	0.326
0.5 - 0.3	0.431	0.120	32	3.60	**0.009**
0.4 - 0.3	0.215	0.098	32	2.19	0.208

2.2. Table S5: Stimulus contrasts

contrast	Estimate (a.u.)	SE	df	t.ratio	p.value
PinkNoise - Applause	-0.399	0.147	32	-2.72	**0.028**
PinkNoise - DentistDrill	-0.734	0.146	32	-5.02	**<0.001**
Applause - DentistDrill	-0.334	0.136	32	-2.45	0.050

2.3. Table S6: Distance contrasts BY Stimulus

contrast	stimulus_label	Estimate (a.u.)	SE	df	t.ratio	p.value
0.6 - 0.7	PinkNoise	-0.359	0.187	32	-1.91	0.331
0.6 - 0.5		0.367	0.192	32	1.91	0.331
0.6 - 0.4		0.235	0.178	32	1.32	0.683
0.6 - 0.3		0.542	0.186	32	2.91	**0.047**
0.7 - 0.5		0.726	0.147	32	4.94	**<0.001**
0.7 - 0.4		0.593	0.144	32	4.11	**0.002**
0.7 - 0.3		0.900	0.173	32	5.20	**<0.001**
0.5 - 0.4		-0.133	0.153	32	-0.86	0.908
0.5 - 0.3		0.174	0.162	32	1.08	0.816

contrast	stimulus_label	Estimate (a.u.)	SE	df	t.ratio	p.value
0.4 - 0.3		0.307	0.130	32	2.37	0.150
0.6 - 0.7	Applause	-0.768	0.177	32	-4.33	**0.001**
0.6 - 0.5		0.093	0.201	32	0.46	0.990
0.6 - 0.4		0.241	0.235	32	1.03	0.842
0.6 - 0.3		0.865	0.204	32	4.25	**0.002**
0.7 - 0.5		0.860	0.184	32	4.69	**<0.001**
0.7 - 0.4		1.009	0.212	32	4.76	**<0.001**
0.7 - 0.3		1.633	0.218	32	7.48	**<0.001**
0.5 - 0.4		0.148	0.202	32	0.73	0.947
0.5 - 0.3		0.773	0.223	32	3.47	**0.012**
0.4 - 0.3		0.625	0.188	32	3.31	**0.018**
0.6 - 0.7	DentistDrill	-0.242	0.207	32	-1.17	0.767
0.6 - 0.5		0.349	0.156	32	2.24	0.191
0.6 - 0.4		0.978	0.187	32	5.22	**<0.001**
0.6 - 0.3		0.693	0.183	32	3.78	**0.005**
0.7 - 0.5		0.591	0.193	32	3.06	**0.034**
0.7 - 0.4		1.221	0.191	32	6.40	**<0.001**
0.7 - 0.3		0.935	0.195	32	4.81	**<0.001**
0.5 - 0.4		0.629	0.146	32	4.30	**0.001**
0.5 - 0.3		0.344	0.187	32	1.84	0.368
0.4 - 0.3		-0.285	0.138	32	-2.07	0.257

2.4. Table S7: Stimulus contrasts BY Distance

contrast	distance	Estimate (a.u.)	SE	df	t.ratio	p.value
PinkNoise - Applause	0.6	-0.328	0.211	32	-1.56	0.278
PinkNoise - DentistDrill		-0.932	0.190	32	-4.91	**<0.001**
Applause - DentistDrill		-0.604	0.234	32	-2.58	**0.038**
PinkNoise - Applause	0.7	-0.738	0.205	32	-3.59	**0.003**
PinkNoise - DentistDrill		-0.816	0.217	32	-3.77	**0.002**

contrast	distance	Estimate (a.u.)	SE	df	t.ratio	p.value
Applause - DentistDrill		-0.078	0.250	32	-0.31	0.947
PinkNoise - Applause		-0.603	0.244	32	-2.47	**0.049**
PinkNoise - DentistDrill	0.5	-0.950	0.212	32	-4.48	**<0.001**
Applause - DentistDrill		-0.348	0.197	32	-1.77	0.197
PinkNoise - Applause		-0.322	0.201	32	-1.61	0.258
PinkNoise - DentistDrill	0.4	-0.188	0.185	32	-1.02	0.571
Applause - DentistDrill		0.134	0.177	32	0.76	0.732
PinkNoise - Applause		-0.005	0.225	32	-0.02	1.000
PinkNoise - DentistDrill	0.3	-0.781	0.222	32	-3.51	**0.004**
Applause - DentistDrill		-0.776	0.200	32	-3.88	**0.001**
